# Versatile Potential of Photo-Cross-Linkable Silk Fibroin: Roadmap from Chemical Processing Toward Regenerative Medicine and Biofabrication Applications

**DOI:** 10.1021/acs.biomac.3c00098

**Published:** 2023-06-23

**Authors:** Jhaleh Amirian, Jacek K. Wychowaniec, Ehsan Amel Zendehdel, Gaurav Sharma, Agnese Brangule, Dace Bandere

**Affiliations:** † Riga Stradins University, Department of Pharmaceutical Chemistry, Riga, LV-1007, Latvia; ‡ Baltic Biomaterials Centre of Excellence, Headquarters at Riga Technical University, Riga, LV-1048, Latvia; § AO Research Institute Davos, Clavadelerstrasse 8, 7270 Davos, Switzerland; ∥ The Faculty of Art and Architecture, Eshragh Institute of Higher Education, F8FQ+9V3 Bojnord, Iran; ⊥ College of Materials Science and Engineering, Shenzhen Key Laboratory of Polymer Science and Technology, Guangdong Research Center for Interfacial Engineering of Functional Materials, Nanshan District Key Laboratory for Biopolymers and Safety Evaluation, Shenzhen University, Shenzhen 518055, P.R. China; # School of Chemistry, Shoolini University, Solan, Himachal Pradesh 173229, India

## Abstract

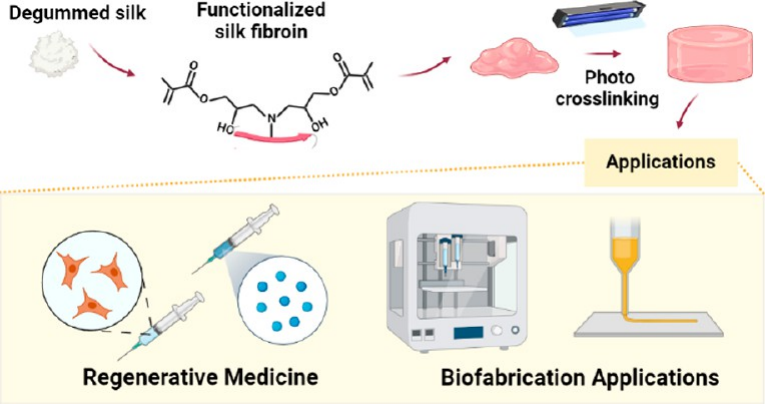

Over the past two decades, hydrogels have come to the forefront of tissue engineering and regenerative medicine due to their biocompatibility, tunable degradation and low immunogenicity. Due to their porosity and polymeric network built up, it is possible to incorporate inside drugs, bioactive molecules, or other biochemically active monomers. Among biopolymers used for the fabrication of functional hydrogels, silk fibroin (SF) has received considerable research attention owing to its known biocompatibility and tunable range of mechanical properties. However, its relatively simple structure limits the potential usability. One of the emerging strategies is a chemical functionalization of SF, allowing for the introduction of methacrylate groups. This allows the versatile processing capability, including photo-cross-linking, which makes SF a useful polymer as a bioink for 3D printing. The methacrylation reaction has been done using numerous monomers such as methacrylic anhydride (MA), 2-isocyanatoethyl methacrylate (IEM), or glycidyl methacrylate (GMA). In this Review, we summarize the chemical functionalization strategies of SF materials and their resulting physicochemical properties. More specifically, a brief explanation of the different functionalization methods, the cross-linking principles, possibilities, and limitations of methacrylate compound functionalization are provided. In addition, we describe types of functional SF hydrogels and link their design principles to the performance in applications in the broad fields of biofabrication, tissue engineering, and regenerative medicine. We anticipate that the provided guidelines will contribute to the future development of SF hydrogels and their composites by providing the rational design of new mechanisms linked to the successful realization of targeted biomedical application.

## 1 Introduction

In recent years various types of hydrogels based on natural and synthetic polymers were employing multiple different cross-linking mechanisms.^1−3^ It has been shown that the appropriate choice of chemical cross-linking approach leads to the generation of hydrogel networks with properties tailored to the desired clinical or tissue engineering application.^4−6^ Among all types of hydrogels, peptide- and protein-based hydrogels have been widely studied as biocompatible matrices for numerous biomedical applications.^2,7−12^ These molecules typically form safe and effective biomaterials due to their ability to form stable networks under mild conditions, their excellent intrinsic biocompatibility, as well as tunable biochemical and biophysical properties.^1,2,5,8−12^ Their physical and biochemical properties depend on the composition, the polymerization method, and the type and degree of cross-linking.^5^ The utilization of cross-linking approaches has led to the development of many applications for these hydrogels in biomedical science, especially in designing injectable cell and drug cargo vehicles.^1,6,10^ Moreover, they have become an integral part of 3D culture research to investigate the cellular proliferation and migration as well as interactions between encapsulated cells (cell–cell) and cells with the matrix (cell–material).^8,10^ Since they mimic the structure and characteristics of extracellular matrix (ECM), they appear as ideal mimicking biomaterials for many biomedical applications.^1,2,6,11,12^

Silk fibroin (SF) is a protein-based biopolymer that shows good biocompatibility, adjustable biodegradability, and low immunogenicity.^13,14^ These characteristics make it suitable to form hydrogels for the use as a versatile tissue engineering support. It has been reported that SF has been used as scaffolds/fibrous membranes,^15^ hydrogels,^16^ in animal models for bone^17^ and cartilage tissue engineering,^18^ wound healing,^16,19^ and as hemostatic agents.^20^ On the other hand, many studies have shown that the regenerated SF materials still lack suitable mechanical properties for the targeted application.^21^ It should be noted that degumming process conditions can significantly change the physicochemical and mechanical properties of silk fibers when silk sericin (SS) is removed completely.^22^ Various approaches have also been employed to use SF as a bioink,^23,24^ including enzymatic cross-linking, chemical modifications, and compositing with other materials.^25^

Here we present a review of recent studies on the synthesis, characterizations, and biomedical applications of photo-cross-linkable silk fibroin-based materials synthesized by the introduction of methacrylic anhydride (MA),^26^ glycidyl methacrylate (GMA),^27−34^ 2-isocyanatoethyl methacrylate (IEM),^35−38^ carbic anhydride (CA),^39,40^ and norbornene (NB) to create photo-cross-linkable materials. Often these are reported and abbreviated as methacrylated silk fibroin (SFMA), methacrylated silk fibroin sealant (Sil-MAS), silk fibroin methacrylate (SF-IEM), norbornene-functionalized silk fibroin (SF-NB), or silk fibroin methacrylate (SF-GMA). Based on the fact that SFMA is a derivative of silk fibroin, we suggest “methacrylated silk fibroin” would be the most appropriate name, which also matches the widely accepted abbreviation SFMA.

As a result of photoinitiated radical polymerization, SFMA forms hydrogels with covalently bonded networks. SFMA hydrogels, in the presence of a photoinitiator, can be covalently cured by directly exposing them to UV or visible light. Based on studies previously conducted, SFMA shows greater mechanical strength in comparison to other types of methacrylated hydrogels, such as gelatine methacrylate (GelMA).^5,24^ Apart from its ability to covalently bond when exposed to UV light or visible light, the mechanical properties of the SFMA also come from the physical entanglements of SF molecules through the conformational interactions of its β-sheet.^41^ Photopolymerization can occur at mild conditions (room temperature, neutral pH, aqueous environments, etc.) and can be regulated spatiotemporally.^29,33,34^ Therefore, SF provides opportunities as an ideal platform for manipulating cellular behavior, studying the interaction of cells and engineering tissues using bio- or microfabrication approaches.

The objective of this Review is to provide an overview of recent studies related to SFMA hydrogel synthesis, characterization, and formation of its composites with other widely used materials. Here, we (1) review the recent studies related to the SF degumming and extraction methods, and present their advantages and disadvantages; (2) review the current trends for modification of the SF for the preparation of the photo-cross-linkable bioinks based on SF; (3) discuss the latest methodology proposed for microfabricating the SFMA hydrogel as well as the benefits of resulting SFMA-based biomaterials; and (4) summarize the most recent developments in SFMA-based hydrogels in different aspects of tissue engineering and regenerative medicine and link their performance to the structural and fabrication aspects discussed with respect to points 1–3.

### 1.1 Silk Protein Source and Structure

**Figure 1:**
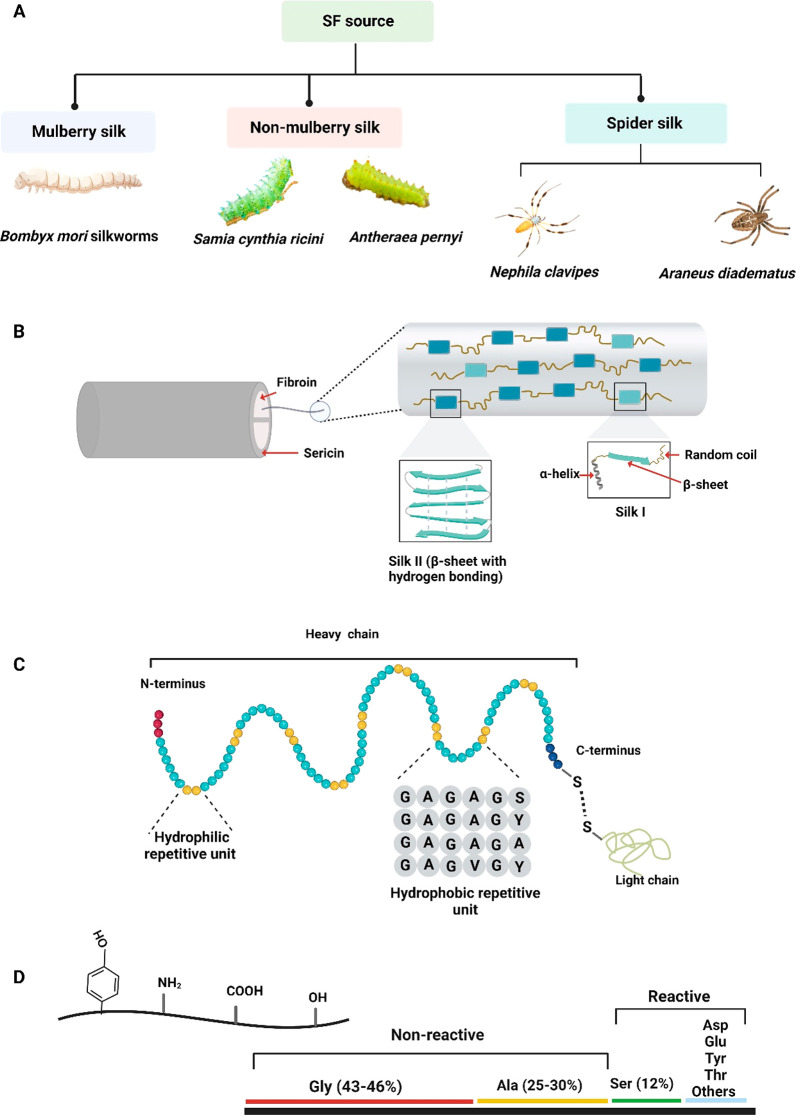
An illustration of (A) silk’s natural source, (B) raw silk fibers consist of fibroin fibers coated with sericin before and after degumming, representative fibroin polymers consisting of silk I and silk II, (C) SF H- and L-chains with hydrophilic and hydrophobic units at the N- and C-terminus, and (D) amino acids of the H chain of SF with reactive and nonreactive portions, along with representative functional groups present in SF for chemical modification and cross-linking figure created with Biorender.com.

Silk is produced from various sources, including silkworms, spiders, and mites from the phylum *Arthropoda*, and materials produced from all of these have received a lot of attention in tissue engineering and regenerative medicine.^42−44^ Some of the most common types are shown in Figure 1A and include the following:

I. SF is derived from *Bombyx mori* silkworms. It is also known as mulberry silk. They were also used in the textile industry because they could be produced in large quantities.^43^
II. The SF derived from other animals (nonmulberry silk) is characterized by polyalanine repeats throughout the crystal structure. Nonmulberry silk includes *Antheraea mylitta* (tasar), *Antheraea assamensis* (muga), *Antheraea pernyi*, *Samia cynthia ricini* (eri), and others.^45^ Silk from the silk moth *Antheraea assamensis* from India is unique among all kinds because it also contains RGD adhesive binding epitopes missing in the mulberry silk.^43^
III. There are only genetically engineered options for dragline silk derived from spiders such as *Nephila clavipes* and *Araneus diadematus*.^43,46^ Silk spiders are light, strong, elastic, and have high mechanical properties comparable to some polymers, such as kevlar.^46−48^

Silk is primarily consisting of SF and SS, with SF being the main component of the material, typically constituting about 75% of it (Figure 1B).^49^ Silk is one of the strongest fibers known to nature, with a tensile strength of up to 4.8 GPa and a density of 1.3 g/cm^3^.^50^ It consists of repetitive hydrophobic modules and small hydrophilic groups (Figure 1C).^51^ A part of the repetitive hydrophobic part of the sequence is composed of amino acids with short side chains, including glycine (Gly) and alanine (Ala). In contrast, the hydrophilic groups are composed of larger side chain polar amino acids.^52^

The SF contains different functional groups, such as hydroxyl, carboxyl, and amine groups (Figure 1D), which can be further reacted with other chemical species, e.g., using coupling reactions.^24^ There are three major amino acids found in SF from *Bombyx mori*, which are Gly (43–46%), Ala (25–30%), and Ser (12%). SF contains over 5000 amino acids, including reactive and nonreactive amino acids. On the basis of previous research, SF contains 1105 of the 5000 reactive amino acids.^24^ While Gly and Ala are nonreactive, serine (Ser), threonine (Thr), aspartic acid (Asp), glutamic acid (Glu), and tyrosine (Tyr) are reactive parts of the SF that can all be easily chemically modified (Figure 1B,D).^53^ It was found that an amorphous and crystallized region exists within the structure of SF. Foremost, these crystalline structures consist predominantly of β-sheet structures, with a dominant conformation of β-turns (referred to as silk I) and a folded β-sheet structure (referred to as silk II), schematically depicted in Figure 1B.^54^ While the exact secondary composition of SF consist predominantly of β-sheets, lower amounts of secondary structures, including α-helices (primarily 3_1_-helices), and an unordered structure exist and are typically found in the silk I region. The exact proportion of each secondary structure depends on the original source of SF, as well as the processing method and environment it is placed in (e.g., salt content or type of solvent).^54,55^ Originally, SF was referred to as an α-form at an early stage to distinguish it from the β-form (silk II). Therefore, to avoid confusion between the α-form and the α-helix, the term silk I was introdocued for SF before spinning. However, there are a number of papers that suggest that the structure of silk I consists partly of α-helices based on IR and Raman spectra.^54−57^ On the other hand, several studies identified the silk I structure as a type II β-turn, which was thoroughly confirmed using a variety of solid-state NMR and solution NMR techniques along with carefully chosen stable isotope-labeled model peptides.^58^ Vass et al. came to the conclusion that the silk I structure should predominantly consist of a type II β-turn with a minor abundance of α-helices,^59,53^ hence, our representation in Figure 1C, stemming from different predominant conformations noted.^60^ Silk I has a metastable form, which is water-soluble, whereas silk II has the most stable structures and is not soluble in water.^60^ Silk II and SF chains are linked by hydrogen bonds; the β-sheet motif of SF is a physically cross-linked material that connects together SF molecules into a 3D network, ensuring the strength and durability of the formed hydrogels.^60^ In contrast, amorphous structures that include α-helices, random coils, and type II β-turns typically have a lower stability than crystalline structures.^60^ As SF consists of hydrophobic as well as hydrophilic regions, the hydrophilic regions are responsible for the material’s water solubility, elasticity, and toughness.^60^ Hydrophobic regions have an ability to form intermolecular interactions, resulting in a conformational change from random-coils or α-helix to a β-sheet motif, depending on the external environmental conditions.^60,61^ SF consists of a heavy (H)-chain of approximately 350 kDa and a light (L)-chain of approximately 25 kDa, which are bridged by a disulfide bond, as depicted in Figure 1D.^47,52,62^

### 1.2 SF Extraction Method

**Figure 2:**
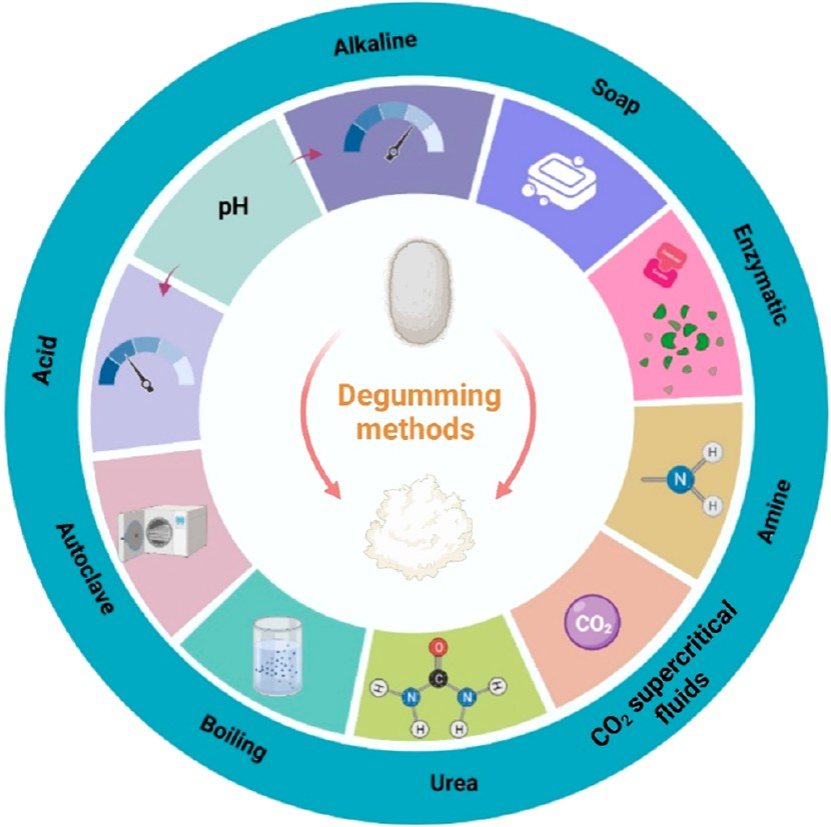
Illustration of an alternative degumming technique for silk. Figure created with Biorender.com.

Extracting SF from silk glands or silkworm cocoons can be accomplished in several ways. A key step in this process involves removing sericin from the cocoons (“degumming”) and subsequently dissolving and purifying them.^63,64^ There are numerous ways that the SF can be degummed, including by acids,^63,65^ alkalines,^63^ soaps,^63,65^ amines,^63,66^ enzymes,^63,67^ urea,^68^ sodium carbonate,^64,65^ ultrasonication,^63,69^ autoclaving,^68^ carbon dioxide (CO_2_) supercritical fluids,^63,70^ and boiling,^71^ all as shown in Figure 2. In Table 1, we provided a list of some of the advantages and disadvantages of each degumming approach. When degumming is being carried out in the textile industry, boiling water or detergent solutions are used as the method of cleaning. However, as part of these methods of removing sericin in the laboratory, boiling in sodium carbonate and autoclaving are among the most widely used.^47^ Studies have indicated that sodium carbonate dissolved in boiling water is the most commonly used method for degumming silk for biomedical applications. Silk fibers which have been degummed are generally dissolved in inorganic salts such as high concentrations of LiCl,^72^ LiBr solution,^73,74^ NaSCN solution,^75^ ZnCl_2_ solution,^76^ or in a ternary solvent mixture comprising CaCl_2_/ethanol/water.^47,64,68,77^ Salts such as these are also capable of breaking hydrogen bonds, ultimately disassembling the β-crystalline structure of degummed silk. Purification is carried out by dialysis against water and removing salts and impurities in order to obtain pure SF.^64^ Moreover, SF can be extracted and stored in lyophilized form for long-term storage and, later on, can be dissolved in organic solvents such as HFIP (1,1,1,3,3,3-hexafluoro-2-propanol)^78^ and formic acid^79^ to produce SF scaffolds in different forms including fibers, gels, or sponges.

**Table 1:** Comparison between Common Degumming Methods for SF

degumming method	reagents	advantages	drawbacks	ref
alkaline degumming	Na_2_CO_3_	strong and effective action	possibility of fibroin damage	(69,80)
	NaHCO_3_	fast procedure	decreasing fibers strength	
	Na_2_HPO_4_	more cost-effective	β-sheet structure severely damaged	
	Na_3_PO_4_	lowering molecular weight	time consuming	
	Na_2_[B_4_O_5_(OH)_4_]·8H_2_O	a simple approach	sericin degradation	
acid degumming	tartaric acid	improvement in tensile strength	slight decrease in dye uptake	(69,81)
	lactic acid			
	citric acid		restricted hydrolysis	
	oxalic acid	the degumming bath can be reused		
	malonic acid			
	succinic acid			
	trichloroacetic acid			
	dichloroacetic acid			
	monochloroacetic acid			
	glacial acetic acid			
soap degumming	marseille, sodium laurate, sodium myristate, sodium stearate	whitening and brightening silk	the degumming bath is not reusable	(63,82)
		excellent strength, elasticity	effluent problems	
		avoids fiber coagulation	metal ions in water can form insoluble metal soaps on silk	
enzymatic degumming	papain	eliminates uneven dyeing	simple enzyme deactivation	(67,71)
	trypsin			
	proteases	enhances dye affinity (especially with reactive dyes)	expensive	
		completely removes sericin	risk of overreaction with fibers	
		environmentally friendly	not suitable for large-scale commercial production	
		works at a moderate pH and temperature	not efficient in removing hydrophobic impurities	
amine degumming	methylamine	providing undamaged and uniform silk	not easily applicable to industry	(63,66)
	ethylarnine	fibers	low degumming rate	
	diethylamine	water does not affect degumming		
	triethylamine	whitening and brightening silk	unpleasant odors	
	marseilles soap	shorter processing times		
		lower temperature and less time required than soap degumming		
carbon dioxide supercritical fluid degumming	CO_2_	maintains the purity of sericin as well	high costs and processing demand of high-tech equipment	(70)
		contamination-free		
		reduced amount of wastewater		
		low energy consumption		
degumming by urea		high reproducibility	undesirable degradation of SF	(83)
		SS produced by urea degumming has higher antityrosinase activity than SS produced by Na_2_CO_3_ degumming		
high temperature and pressure (autoclaving)		highly efficient	removing only the outer layer of sericin	(68,71)
		no contamination	effects absorbency and whiteness	
		without contamination		
		cost-effective method		
ultrasonication		environmentally friendly	adding soap, alkali, acid, or enzyme is required	(69,84)
		improved energy efficiency		
		reduced chemical usage	utilization of electrical energy for acoustic purposes	
		increasing productivity		

## 2 Chemical Modification of the SF for Introducing the Methacrylate Group

There are numerous techniques used to introduce functional methacryloyl groups into SF, which render SF a photo-cross-linkable polymer.^85^ There are primarily two ways for photopolymerizing SF-based macromonomers. As a first method, the reducing agents (for example, ruthenium complexes) can be used to photopolymerize pristine SF, resulting in covalent bond formation.^86,87^ This approach uses for example the presence of tyrosine on pristine SF, which can easily form dityrosine bonds when catalyzed with a ruthenium complex under light exposure. A second approach involves prechemical modification of SF protein, where active amino acids are used for the grafting of other functional groups, such as methyl methacrylate,^88^ vinyl acrylate,^89^ and methacrylic acid derivatives.^5,85,90,91^ Consequently, modified SF chains are photopolymerized in the presence of the photoinitiator under UV or visible light irradiation. Using the second approach, which is the main objective of the current review, the modified SF can be precisely tuned in terms of its physicochemical properties, chemical strength, hydrophilicity, and swelling behavior in order to meet the needs of specific tissue engineering applications. SF has an amino acid sequence that consists of approximately 12% serine, 0.3% arginine, and 0.2% lysine.^24,26,53,92^ Nucleophilic hydroxyl groups are weakly present in serine residues, while amino groups are strongly present in lysine and arginine residues.^26^ As a result, covalent bonds may form between these functional groups and reactive electrophiles, such as IEM.^26^ Hence, a chemically modified SF derivative can be produced.^90^ Free-radical polymerization can be readily performed by the grafting of materials containing double C=C bond groups, such as methacryloyl groups, on polymer chains. Several studies have been conducted based on the preparation of the photo-cross-linkable SF by incorporating the MA,^26,93^ and GMA,^24,86^ and IEM, on the SF chain. Depending on the conditions and modification reagents, methacryloyl substitution can be carried out at its amine, hydroxyl, and carboxyl groups. Until now, the components used to introduce methacryloyl groups on SF are MA, GMA,^86^ and IEM. However, these chemicals have been shown to induce different effects on SF properties. In the process of methacrylating SF by MA, a byproduct known as methacrylic acid is formed, resulting in a reduction of pH and crystallization of SF. A methacrylation of SF by GMA is based on an epoxide ring opening, and this type of reaction does not produce any byproducts, causing a reduction of pH, making this a more facile type of methacrylation. MA and GMA are both proposed to react with the primary amines of SF that constitute around 0.2% of its total amino acid content.^86,94^ Among typical reactive amino acids, the primary amino acid groups are lysine 0.2%, arginine 0.3%, asparagine 0.4%, and glutamine 0.2%, with lysine being the predominant amino acid participating in MA and GMA reactions. GMA is also capable of reacting with carboxyl and hydroxyl groups which are present in SF.^12,24,53,95^ Based on a recent report, GMA can react with carboxyl and hydroxyl groups either via transesterification or opening of the epoxide ring.^96,94^ The SF heavy chain contains a higher molar ratio of hydroxyl and carboxyl groups, approximately 19.4%, and a lower molar ratio of primary amine groups (lysine 0.2%). As a result, methacryloyl substitution on the carboxyl and hydroxyl groups may lead to an increase in cross-linkable sites in SF, meaning an increase in photo-cross-linking density, which can be reflected in the final mechanical properties of the formed hydrogels. In subsequent sections, we provide a detailed analysis of these reactions.

### 2.1 How Modification Reactions are Performed

**Figure 3:**
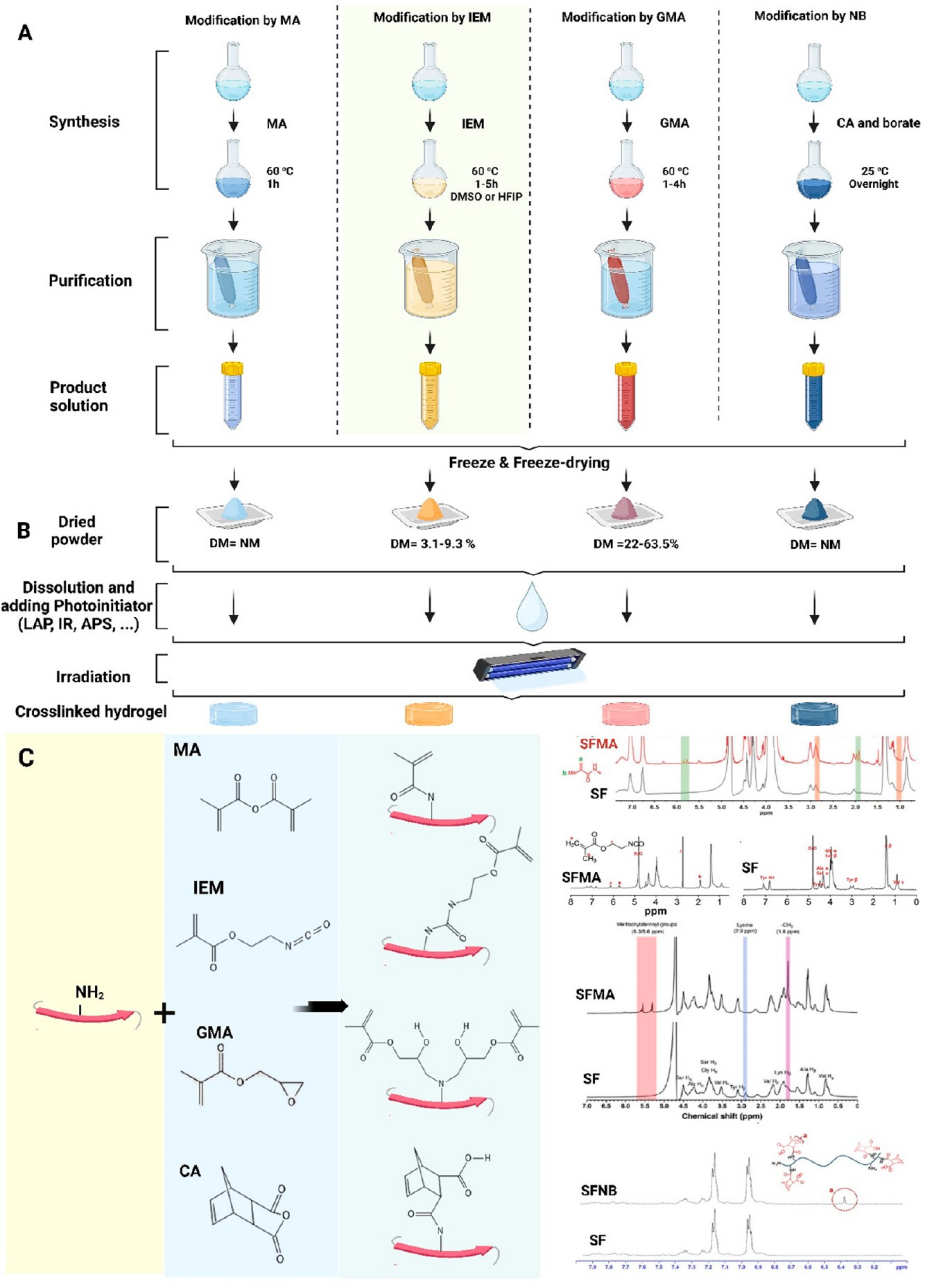
(A) Diagrams of various approaches for modifying the SF with monomers containing double bonds, including MA, IEM, GMA, and NB, to produce photo-cross-linkable SF materials, (B) achieved methacrylation by each method (NM: not mentioned), and (C) SFMA chemical reactions and their representative NMR compared to SF. Adopted with permission from ref (93). Copyright 2021 Wiley-VCH GmbH. Adopted with permission from ref (35). Copyright 2018 Elsevier Ltd. Adopted with permission from ref (32). Copyright 2021 American Chemical Society. Adopted with permission from ref (39). Copyright 2016 The Royal Society of Chemistry. Except the adopted NMR spectra with listed copyright permissions, figure was created with Biorender.com.

In Figure 3 we schematically depict all the most important methacrylation reactions, providing basic information about the methacrylation agents that are used, typical conditions, as well as providing exemplar NMR spectra, confirming successfully utilized reactions. In the following sections, we discuss each type of reaction in more detail.

#### 2.1.1 Modification by MA

The MA has been used in numerous studies for the methacrylation of gelatin (i.e. to synthesize GelMA).^5,97^ The MA is a relatively inexpensive and stable methacrylating agent compared e.g. to the IEM, and can be used in aqueous solutions, which overall makes it a primary choice of methacrylation agent.^26^ Bessonov et al. reported the first instance of methacrylation of the SF with MA in 2019.^26^ By this method, SFMA is synthesized by direct reaction of SF with MA in a potassium phosphate buffer solution (at pH = 7.0) at 50 °C for 1 h under vigorous stirring.^26^ With this reaction, the methacryloyl groups are introduced on the reactive amines of the residues of amino acids present in SF. Noting that the total amount of amine in SF is approximately 0.2%, this is a maximum limit of the degree of methacrylation, overall being low compared to many other biopolymers.^98,99^ Nevertheless, we expect the possibility to modulate it within this range by other factors, such as controlling temperature or the total time of the reaction. So far only two studies focused on the methacrylation by means of MA, however, no information has been provided on the final achieved degree of methacrylation.^26,93^ Consequently, it is possible the minor variation in the degree of methacrylation in this range may result in SFMA products with lower number of cross-links and thus variable mechanical properties when subsequently photo-cross-linked. This variation however has not yet been reported, as degrees of methacrylation in the two reports were not provided. A stop-reaction is accomplished by diluting the reaction mixture (roughly two times) with potassium phosphate buffer solution, followed by several days dialysis against water through cellulose cutoff tubing to remove residual impurities, including unreacted MA, methacrylic acid, and other byproducts of the reaction (Figure 3A). As a final step, the dialyzed solution is frozen and lyophilized for long-term storage of a product reconstituting solid (Figure 3B).^26^ However, MA has not been extensively employed for methacrylation of the SF due to the resulting lower degree of methacrylation and reported reduction of efficiency of the reaction caused by the decrease of pH from the resulting methacrylic acid byproduct.

#### 2.1.2 Modification by IEM

IEM is one of the methacrylation reagents, which is used for methacrylation of SF.^38^ Since IEM is an electrophilic compound, it is capable of reacting with both weak hydroxyl groups and strong nucleophiles (amino groups in lysine and arginine residues) at comparable rates.^26^ Unlike the MA reaction, which can be conducted in an aqueous medium, methacrylation by IEM should be carried out in anhydrous dimethyl sulfoxide (DMSO).^26^ A study by Kurland and colleagues reports the synthesis of silk fibroin-based photoresist materials by the use of IEM.^37^ A general way to conduct this type of reaction is to dissolve SF in a solution that contains 1 M lithium chloride dissolved in dimethyl sulfoxide (LiCl/DMSO; Figure 3A)^35,37,38^ or HFIP.^36^ Afterward, IEM was added, and the reaction was stirred for 1–5 h in a nitrogen gas atmosphere at 60 °C. For various methacrylation degrees, different amounts of IEM, between 0.25 and 2.0 mM per gram of SF, were added to the solution. Upon stopping the reaction by adding 10 X dilution of deionized (DI) water, solution was dialyzed at room temperature for 3 days with a cellulose acetate membrane (MWCO: 12–14 kDa). In the next step, the solution was centrifuged and finally freeze-dried (Figure 3A). It is also noteworthy to mention that methacrylation using IEM produces macromers composed largely of urethane-acrylates rather than methacrylamides.^26^ Due to the expanded capability of IEM reacting, higher degrees of methacrylation are obtained than in the case of MA, with values up to 9.3% reported (Figure 3B).^35^

#### 2.1.3 Modification by GMA

**Figure 4:**
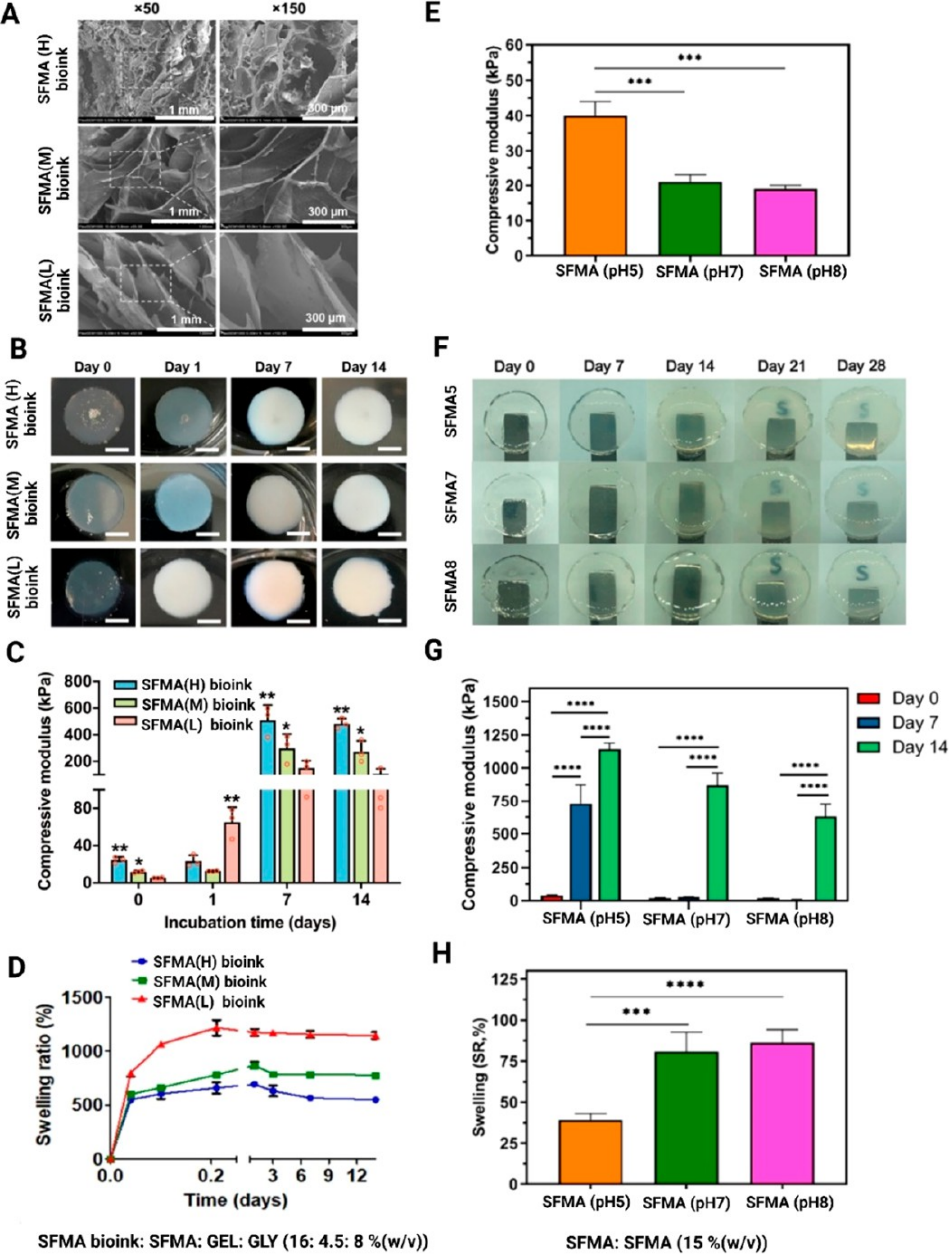
Material characterization of SFMA bioink (SFMA: GEL: GLY (16:4.5:8% (w/v)) constructs with different degrees of methacrylate (H: high, M: moderate, and L: low): (A) scanning electron micrographs, (B) changes in the conformation during incubation at 0, 1, 7, and 14 days, (C) compressive elastic moduli after 0, 1, 7, and 14 days incubation, (D) swelling ratio of hydrogel after 14 days. Adopted with permission from ref (27). Copyright 2020 American Chemical Society. Representative material properties of SFMA (15%) hydrogel at three different pH levels: (E) compressive moduli, (F) hydrogel conformational changes during 28 days incubation in PBS, (G) hydrogel toughening effects after 0, 7, and 14 days incubation, and (H) SFMA swelling properties in PBS. Adopted with permission from ref (30). Copyright 2021 American Chemical Society.

Another substance that has recently received a lot of attention for the functionalization of SF is GMA.^12,31,99^ In this method SFMA is synthesized by reacting SF with GMA in lithium bromide with a concentration of 9.3 M at 60–65 °C for 1–4 h under vigorous stirring conditions (Figure 3A).^24,27−33^ The degree of methacrylation will be dependent on the primary amine on lysine and will occur through the ring-opening of epoxy at GMA.^24^ However, based on the recent report, GMA can also react with other groups, such as carboxyl and hydroxyl, either via transesterification or the opening of the epoxide ring. The SF heavy chain contains a higher molar ratio of hydroxyl and carboxyl groups, approximately 19.4%, and a lower molar ratio of primary amine groups (lysine 0.2%). As a result, methacryloyl substitution on the carboxyl and hydroxyl groups may lead to an increase in cross-linkable sites in SF, meaning an increase in photo-cross-linking density (Figure 3B,C). Various concentrations of GMA with respect to SF and stirring (reaction) time are added to achieve different levels of methacrylation. As the methacrylation takes place through the opening of the epoxide ring, there are no byproducts generated that would reduce the pH of the final solution. In 2018, Kim et al. reported this method of methacrylation of SF using GMA in order to prepare 3D-printed bioink for printing highly complex organ structures, such as the ear, vessel, brain, and trachea.^24^ GMA was added in various molar ratios to SFMA to investigate the influence of methacrylation level on final bioink physicochemical properties, structure, and their biocompatibility. Kim et al. synthesized SFMA hydrogels with different methacrylation degrees (22.4, 32.2, 42.0, and 39.2%), using 141, 282, 424, and 705 mM GMA solutions in the reaction, respectively.^24^ Noteworthy, there was no linear relationship noted in this case and highest degree of methacrylation was obtained for 424 mM of GMA. This enables control over how the characteristics of SFMA can be altered through manipulation of its synthesis approaches, methacrylation agents, and processing. In another report by Costa et al., SFMA hydrogel mechanical properties were correlated to a degree of methacrylation.^27^ The SFMA bioink with a higher methacrylation degree, 63.5%, has shown denser and smaller pore size structure, higher mechanical strength, 24.4 ± 2.8 kPa, and lower swelling ratio, as compared to SFMA with lower methacrylation, 44.5%, with larger pores, and lower mechanical strength, 5.4 ± 0.3 kPa (Figure 4A–C). It is obvious that the degree of methacrylation has an effect on the degree of conformation change that occurs after cross-linking with UV light. When a color change occurs, it indicates the transition between a random coil and a β-sheet. SFMA that is highly methacrylated maintains its transparency, while SFMA that is moderate and has a low degree of methacrylation produces an opaque white color within a short period of time (Figure 4B). A high degree of methacrylation results in smaller pores in SFMA, which results in lower swelling ratios for SFMA constructs (Figure 4D).

Barroso et al. investigated how three different pH values (5, 7, and 8) can affect the rheological properties, compressive modulus, and network swelling of SFMA hydrogels synthesized by means of GMA.^30^ The corresponding values for SFMA hydrogels compressive modulus were 40 ± 4 kPa at lower pH (pH 5) in comparison to 21 ± 2 kPa at higher pH (pH 7), as shown in Table 2.^30^ A two-orders of magnitude increase in SFMA compressive modulus (at pH 5) was observed within 14 days, and a one-order of magnitude increase in SFMA compressive modulus was observed at pH 7 and 8, as shown in Figure 4G. The compressive modulus of SFMA hydrogels prepared at pH 7 and 8 were in fact not statistically significant between days 0 and 7. Figure 4H illustrates the swelling ratio for SFMA at pH 7 and 8, which shows significant increases (2-fold) compared to SFMA at pH 5. In summary, this study showed that the swelling characteristics of the hydrogel were directly correlated to the pH.

**Table 2:** Brief Summary of the Type and Concentration of SFMA and Photoinitiator, Wavelength, Intensity, and Time Used for Photo-Cross-Linking in SFMA^a^

	hydrogel % (w/v)				rigidity		
grafted monomer	photoinitiator % (w/v)	photoinitiator	wavelength/irradiation power	time			ref
MA	SFMA/SFMA-Ce6 7.5% and 10%	Irgacure 2959	365 nm	60 s	7.5	326 kPa	(93)
					10	553 kPa	
	0.5%		30 mW/cm^2^				
MA	10% w/v	TPO	UV light	10 min	HFIP	480 kPa	(26)
					FA	120 kPa	
	0.5% w/v		NM				
IEM	2%	Irgacure 2959	365 nm	1.5 s	NM		(36)
	0.6%		2 mW/cm^2^				
IEM	2%	Irgacure 2959	320–500 nm	1 s	0.6	15.6 ± 1.1 GPa	(37)
	0.6%		NM				
IEM	12, 20, 28% (w/w) FFP blended with eumelanin	Irgacure 2959	365 nm	3 s	NM		(38)
	2.5% (w/v)		20 mW/cm^2^				
IEM	10–20 wt %	LAP	365 nm	5 min	NM		(35)
	1 mM		5 mW/cm^2^				
GMA	electrospun fiber	FMN	454 nm	10 min	NM		(34)
	2 mM of FMN						
	50 mM of SPS	SPS	2500 mW/cm^2^				
GMA	25%	LAP	365 nm	10–30 s	NM		(33)
	0.3%		6 mW/cm^2^				
GMA	15%	LAP	365 nm	5 min	pH 5	40 ± 4 kPa	(30)
			3 mW/cm^2^ UV-A		pH 7	21 ± 2 kPa	
					pH 8		
	0.5%		3 mW/cm^2^				
GMA	20%	LAP	365 nm	NM			(32)
	0.2%		1300 MW				
GMA	10 and 20%	LAP	NM	NM			(31)
	5 and 75 mg						
GMA	0.3% or 0.03%	LAP	365 nm	10 min	NM		(29)
	0.2% or 0.04%		NM				
GMA	20%	LAP	NM	200 s			(28)
	0.3%		4.79 mW/cm^2^				
GMA	SFMA:GEL:GLY 16:4.5:8% (w/v)	Irgacure 2959	BlueWave MX–1 50 LED	120 s	14.7 ± 2.1 kPa		(27)
	0.1%		400 mW/cm^2^				
CA	SFNB 1%	LAP	365 NM	5 min			(39)
	1 mM		5 mW/cm^2^				
CA	SFNB: 5–15 wt % DTT (DTT: SFNB with 1–10 to 1:50 mass ratio) 0.05–0.5 wt %	Irgacure 2959	NM	NM	1:10	0.9 Pa	(40)
					1:50	1.1 kPa	

a NM: not mentioned.

#### 2.1.4 Modification by NB

Ryu and co-workers described a new synthesis route for the preparation of norbornene functionalized silk fibroin (SFNB). To carry out the modification, the primary amines of the SF are used as nucleophiles for the reaction with CA, resulting in the formation of amino-linked norbornene (NB; Figure 3). The preparation of SFNB can be summarized as follows: SF is reacted with carbic anhydride in aqueous buffer solutions (pH 9) and borate, which are stirred overnight at room temperature in the dark (Figure 3A). As the last step, the solution is filtered and dialyzed against deionized water at 4 °C for 3 days using cellulose acetate tubes (MWCO: 12–14 kDa; Figure 3B). The prepared SF-NB hydrogel shows dual mode gelation behavior, including chemical and physical cross-linking. This dual mode cross-linking is based on thiol–ene photoclicking chemistry and β-sheet formation of the SF.^39^ Contrary to the methacrylate, the norbornene group exhibits less toxicity to cells and are capable of selectively reacting with thiols.^100^ As a consequence, more selective topological gel networks can be formed, since photopolymerization of the same polymer chains is prevented.^100^ Although thiol–ene photoclicking chemistry is different to that of methacrylated moieties, we briefly summarize this here as an addition to the emerging types of chemistries that is expected to be frequently used in the near future.

### 2.2 Photoinitiators Used for Cross-Linking SFMA

SFMA can be photo-cross-linked using multiple photoinitiators that can be cured under light exposure, most commonly at two wavelengths of either λ = 365 or 405 nm. A number of photoinitiators are frequently used in the cross-linking of SFMA, including 2-hydroxy-1-[4-(2-hydroxyethoxy) phenyl]-2-methyl-1-propanone (Irgacure 2959).^36−38,40,93^ and lithium acylphosphinate salt (LAP).^28−33,35,39^ In aqueous environments, the solubility of Irgacure 2959 in water is much lower (around 5 mg/mL) than that of LAP (around 885 mg/mL), however, these are sufficiently high for the photopolymerization to occur. Other types of photoinitiators were also used, such as diphenyl-(2,4,6-trimethylbenzoyl) phosphine oxide (TPO),^26^ flavin mononucleotide (FMN),^34^ and sodium persulfate (SPS).^34^ There are many factors, such as SFMA concentration, photoinitiator concentration, UV exposure time, and radiation power of the light, that allow tuning of the physical properties of the resulting SFMA hydrogels. We summarized those and their influence on final structural properties in Table 2. The amount of photoinitiator used in SFMA affects the overall cross-linking time, quality, final degradation as well as color of the resulting hydrogels. Bucciarelli et al. have looked at how the percentage of LAP affects the color of SFMA hydrogels.^31^ It is likely that this change in color is related to a cross-linking or reaction occurring between SFMA chains in the presence of LAP. It was observed that UV treatment of SFMA samples with high amounts of LAP (15% w/w) resulted in a photoyellowing effect; however, this effect was not observed with SFMA samples with low amounts of LAP (1% w/w).^31^ It has been reported that the impact of UV radiation on amino acids depends on three factors: time, energy absorbed, and peak-wavelength.^101^ Photoyellowing is primarily related to free radical photooxidation of tryptophan and tyrosine residues which leads to yellow products. In this respect, it could be possible that LAP induces the oxidation of tyrosine and tryptophan residues.^31,101^ It is, however, noteworthy to add that 15% w/w concentrations of LAP are beyond the standard regime of low cytotoxicity and would not be used in any biomedical settings. Concentrations lower than 1% w/w should remain the only viable options in future studies to limit any potential cytotoxicity effects.

### 2.3 Hybrid and Composite Hydrogels Based on Photo-Cross-Linkable SF

Typically, hybrid and composite hydrogels are constructed from the combination of a variety of components that have been chosen to synergize the properties of each component in a single biomaterial.^5^ SFMA has also been used in the form of the composite with other types of the synthetic and natural polymers. Here we summarize composite hydrogels composed of SFMA and other compounds, such as *N*,*N*-dimethylacrylamide (DMAA), tetramethylethylenediamine (TEMED), polyethylene glycol diacrylate (PEGDA), graphene oxide (GO), and inorganic and organic nano- and microparticles, such as methacrylated hollow mesoporous silica microparticles (HMSC-MA), as well as molecularly imprinted silk.

**Figure 5:**
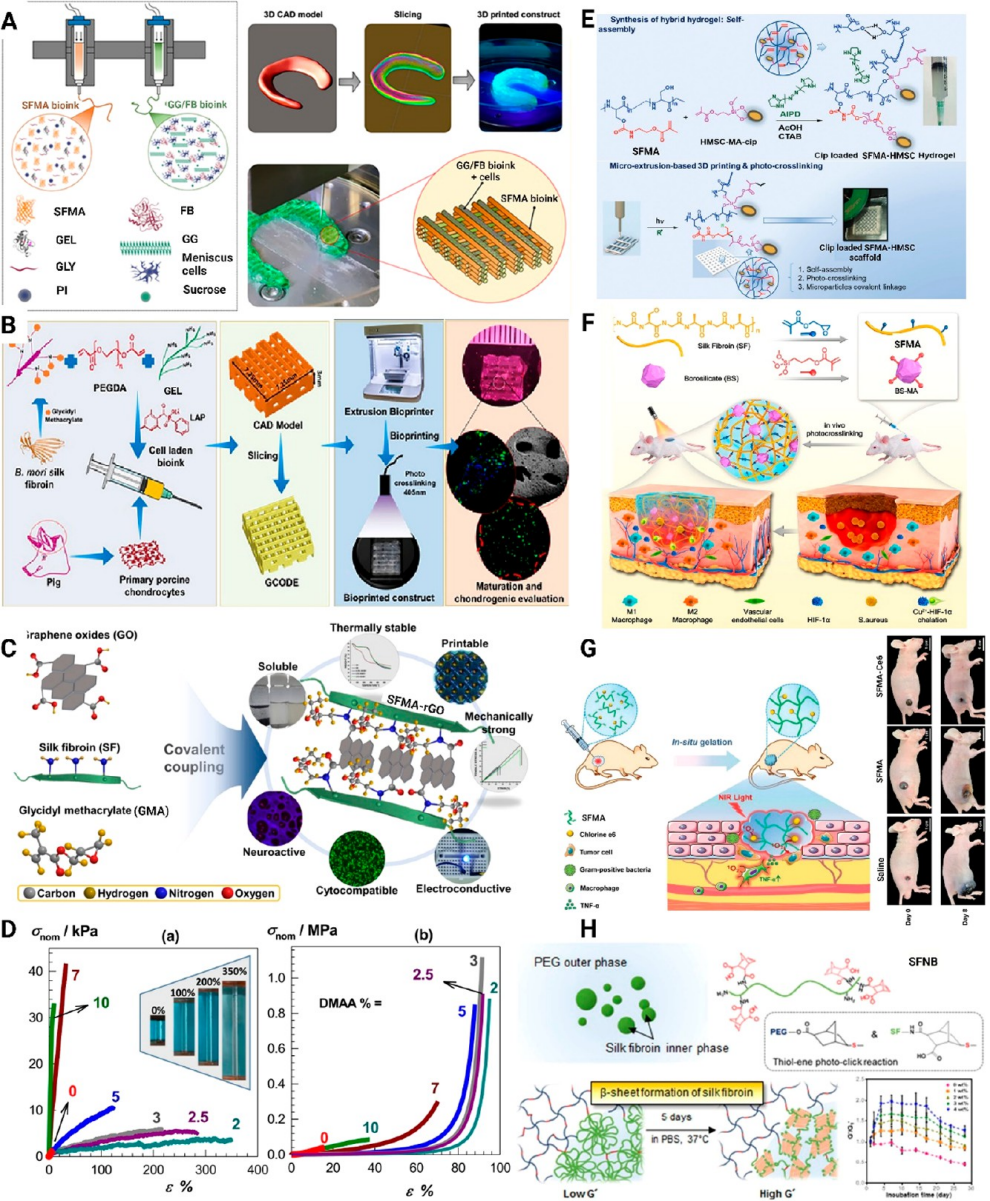
Schematic illustration of (A) the hybrid bioinks made from the SFMA:GEL:GLY B bioink and the GG/FB bioink, as well as the scheme of printing, the 3D CAD model, slicing, and 3D printed constructs. Adopted with permission from ref (27). Copyright 2020 American Chemical Society. (B) 3D bioprinted construct is obtained using extrusion bioprinters and SFMA-PEGDA bioink infused with porcine chondrocytes. Adopted with permission from ref (102). Copyright 2021 Wiley Periodicals LLC. (C) SFMA-GO electroconductive bioinks and their characteristic features. Adopted with permission from ref (28). Copyright 2020 American Chemical Society. (D) Stress–strain curves for hydrogels containing various levels of DMAA (inserted images of SFMA hydrogel containing 2% DMAA during 350% elongation). Adopted with permission from ref (107). Copyright 2020 Elsevier B.V. (E) Homogeneous SFMA gel is generated containing nanoparticles of HMSC-MA and CTAB and microextrusion-based 3D printing simultaneously photo-cross-linking hydrogel constructs. Adopted with permission under Creative Commons CC BY-NC 4.0 License from ref (108). Copyright 2022 Wiley-VCH GmbH. (F) SFMA synthesized with GMA and BS-MA using TMSPMA. Adopted with permission from ref (32). Copyright 2021 American Chemical Society. (G) SFMA-Ce6 Hydrogel for treatment of residual melanoma and wound healing. Adopted with permission from ref (93). Copyright 2021 Wiley-VCH GmbH. (H) Dual mode gelation of SFNB-PEGs with thiol–ene photopolymerization and post gelation of SF molecules by hydrogel structural transition in PBS. Adopted with permission from ref (39). Copyright 2016 The Royal Society of Chemistry.

Costa et al. coprinted porcine primary meniscus cells (pMCs) using gellan gum/fibrinogen (GG/FB) composite bioinks in conjunction with SFMA bioink hydrogels to produce an elastic hybrid construct for advanced fibrocartilaginous tissue regeneration (Figure 5A).^27^ As we have already shown, in this study different levels of methacrylation exhibited different effects on the rheological properties, swelling ratio, and compressive mechanical behavior of the material (Figure 4 A, B, C, and D).

Among the PEG derivatives, PEGDA contains reactive acrylate groups both at the termination ends. This component is widely utilized in the preparation of photo-cross-linkable hydrogels that are cured using light. In addition, PEGDA hydrogels are bioinert, nontoxic, nonimmunogenic, possess tunable physical and chemical properties, and have been shown to be injectable.^102,103^ Bandyopadhyay et al. have developed a bioink made of composite bioink in order to promote cartilage regeneration (Figure 5B).^102^ SFMA, PEGDA, and gelatin have been used as the main components of their bioink. The ratio of SFMA to PEGDA was varied between 2 and 8% (w/v) and 25–100 (w/v), respectively, to determine the best concentrations in order to prepare and print the hydrogels. In addition, 20% (w/v) of gelatin (GEL) was blended into the optimized bioink for improved thermal gelation-based printability as well as shear thinning-based shape fidelity, as well as the retention of the printed layers after deposition. The authors have shown that two compositions, SFMA:PEGDA:GEL (w/v%) of 7:60:20 and 8:60:20, show the best gel formation, consistency, and printability among different compositions. In both of these bioinks, the LAP concentration of 0.2% was used. However, there was no information available about the resulting mechanical properties of each gel.

GO is a two-dimensional nanomaterial obtained from the exfoliation-oxidation of graphite and can be incorporated into hydrogels, such as GelMA,^5,104^ methacrylated chitosan (ChiMA),^105^ and SFMA^28^ to form nanocomposites with enhanced mechanical properties.^5,105,106^ Ajiteru et al. have prepared the composite that is comprised of the SFMA conjugated with graphene oxide (GO), a material that can be utilized as a printable bioink through digital light processing, as shown in Figure 5C. A 0.25% w/v and 2.5% w/v GO was added to the SFMA, resulting in a compressive stress increase to approximately 580 and 770 kPa, which are higher than the pristine SFMA at 550 kPa.^28^ A further increase in conductivity was obtained by adding GO from 0.25 to 2.5% from 0.005 to 0.0065 S/mm, which is greater than the conductivity of SFMA in its original form, 0.0025 S/mm.^28^ Between day 0 and day 3, no significant differences were found between SFMA and SFMA containing 0.25 and 2.5% w/v GO. After 3 days, the proliferation of mouse neuroblastoma (Neuro2a) cells increased progressively; SFMA contains higher levels of proliferation than SFMA with 0.25, 2.5% w/v GO. Comparing the synthesized bioink with conventional SFMA, the synthesized bioink has been shown to demonstrate enhanced mechanical strength, electroconductive properties, and neurogenic properties.^28^

Oral et al. proposed a strategy for the preparation of stretchable SF hydrogels by introducing flexible polymer chains into the brittle SF network, as is shown in Figure 5D. By using this method, it was possible to strengthen the connections between the SF globules. This study reported the use of SFMA and DMAA monomers in combination with TEMED, 1–4-butanediol diglycidyl ether (BDDE), and ammonium persulfate (APS) as cross-linkers, pH-regulators, and initiators, respectively, to construct an interconnected SFMA composite hydrogel network. They found that by incorporation of 2–3% DMMA into the SFMA networks, the brittle hydrogel could be transformed into a stretchable one. A hydrogel containing DMAA had a toughness of 54 kJ/m^3^, which was 20 times greater than one without DMAA, 2.5 kJ/m^3^. Additionally, they have shown that SFMA containing 2% DMAA had a greater elongation ratio, approximately 370%, compared to the control SFMA. As a result, the amount of DMMA incorporated into the SFMA network could be used to tune the final mechanical properties.^107^

Inorganic nanocarriers made of mesoporous silica nanoparticles (MSNs) have been shown promising properties, such as high surface area, large pore volume, easy potential for physical and chemical functionalization, and overall biocompatibility.^109^ Furthermore, due to the large mesopores and hollow interior void, hollow mesoporous silica microparticles have the advantage of carrying a large load of model protein and adjuvant and being useful as a carrier of various biomolecules and drugs.^110^ Ng et al. have developed a photo-cross-linkable microporous aerogel bioink based on methacrylated hollow mesoporous silica microparticles (HMSC-MA) and SFMA (Figure 5E). The scaffold was shown to display a wide range of biophysical and biological properties, including superior mechanical stability and interconnectedness compared to pristine SF scaffolds. A printed scaffold revealed two different ranges of pore sizes, 100–120 μm and 100–1000 μm. A large pore size is a result of voids in structure developed by printing, and a smaller pore size is created by ice replica growth during directional freeze-casting of gel.^108^ According to the results of Ng et al. study, both SFMA and SFMA-HMSC showed cell viability of greater than 95% during a 7-day of cell culture. A positive impact on cell proliferation, infiltration, and osteoblastic differentiation was also observed in SFMA-HMSCs, making them possible biomaterials for the treatment of bone-related diseases.^108^ Pang et al. developed a composition containing SFMA and borosilicate (BS) capable of transforming into a SF-MA-BS hydrogel under UV illumination via MA groups modified on both surfaces (i.e., SFMA and methacrylated BS), Figure 5F.^32^ This composite system can be thoroughly distributed across the entire wound surface and photo-cross-linked *in situ* to form an integral SF-MA-BS hydrogel.^32^ They combined BS and SFMA hydrogel with BS weight percentages of 0, 1, 3, and 6 and cross-linked it under UV light (λ = 365 nm) for 15 s.^32^ The SFMA hydrogel that contained 3% w/v BS had the highest tensile strength when compared with other groups, so they applied this composite system for wound healing.^32^

Tang et al.^93^ recently demonstrated the potential for *in situ* SFMA-based hydrogel and chlorine e6 to enhance wound healing and treatment of cancer. The preparation of SFMA was accomplished by functionalizing SF with MA, as shown in Figure 5G. Additionally, they prepared the SFMA-Ce6 by conjugating the reactive amine of the SFMA to the carboxylic acid functional groups of Chorine e6 (Ce6) using EDC/NHS chemistry. Then, SFMA and SFMA-Ce6 hydrogels were prepared at 7.5 and 10% (w/v) concentrations and their properties, including compressive strength, rheological properties, cytotoxicity, and cell viability were tested. The strength of the SFMA 10% w/v hydrogel is higher than the strength of the SFMA 7.5% w/v, which is 553 and 326 kPa, respectively. The team also evaluated the cytotoxicity of the hydrogel with L929 fibroblasts for 3 days. Both the SFMA and SFMA-Ce6 hydrogels showed higher than 90% cell viability and similar results as the control group. According to their findings, these types of SF based materials have good biocompatibility, over 90%, making them useful in biomedical and wound healing applications. Figure 5G shows that the examined effects of SFMA-Ce6 as a photodynamic therapeutic agent on skin tumors. The hydrogel was placed over the seeded B16F10 melanoma cells on a plate and irradiated under NIR light (20 mW cm^–2^). The viability of the B16F10 melanoma cells is significantly reduced after 10 min of NIR irradiation in the presence of the SFMA-Ce6. It should be noted, however, that the viability of the cells has increased by more than 95% after 10 min of NIR exposure in the presence of the SFMA, indicating hyperthermic and cancer eradication potential of SFMA-Ce6.

The incorporation of norbornene-functionalized 4-arm poly(ethylene glycol) (PEG4NB) in SFNB can also increase the stiffness of the SFNB-PEGNB hydrogel. Ryu et al. developed the SFNB-PEGNB hydrogel, demonstrating dual mode cross-linking as shown in Figure 5H. The SFNB has been used in composite form with PEGNB and cross-linked based on thiol–ene photoclick chemistry. In this study, PEG4NB was mixed with dithiothreitol (DTT), ∼0.03% LAP, where all components were vortexed for 10 s to produce PEG precursor solutions. In the next step, the solution was exposed to 5 mW/cm^2^, 365 nm UV light for 2 min. Incorporating 4% of SFNB into 3, 4, and 5% of PEG4NB resulted in hydrogel shear elastic moduli of approximately 1700, 2750, and 3650 Pa as compared to hydrogels without SFNB that had elastic moduli of 625, 1500, and 2600 kPa, respectively.^39^ Additionally, they observed that SFNB-PEGNB hydrogel stiffness increased over a 5 days period, and the gel modulus remained the same for 2 weeks following the gel preparation. It was also demonstrated that the inclusion of 4% SFNB microgels in PEG-NB hydrogels resulted in a 2-fold increase in shear modulus compared to the modulus on day one after gelation.^39^

This section comprised discussing selected examples of various materials composited with SFMA or SFNB and how they affected both physicochemical and biological properties of resulting materials. In next section, we will discuss potential for fabricating various structures based on SFMA hydrogels and briefly summarize their uses.

## 3 Microfabrication and Nanofabrication of SFMA Hydrogels

**Figure 6:**
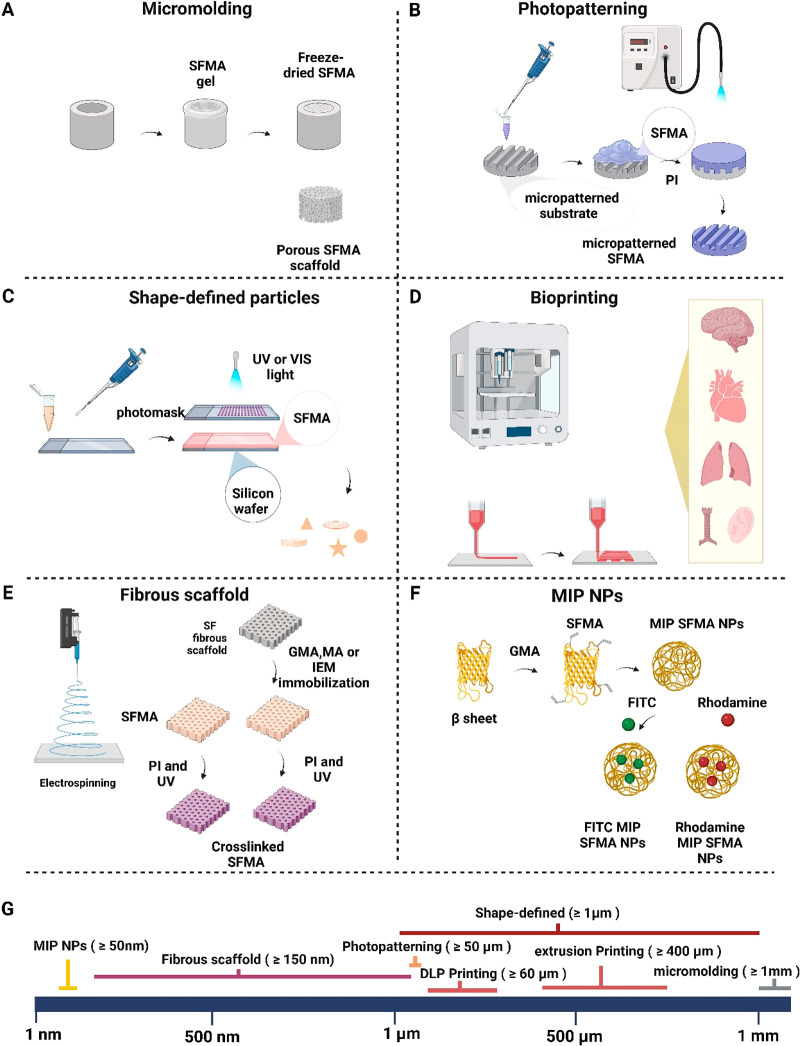
(A–F) An overview of the micro- and nanofabrication techniques used to fabricate SFMA hydrogel constructs, and (G) resolution and size achieved by each method. Figure created with Biorender.com.

The growing interest in additive manufacturing and biofabrication technologies has paved the way for the synthesis and chemical modifications of several hydrogel forming biopolymers,^111,112^ including collagen, gelatin, hyaluronan, SF, chitosan, alginate, pectin, dextran, and PVA. The manufacturing of tissue constructs and ECM mimics is, however, still a challenge.^113^ The matrices that are built to simulate native ECM should be able to support cell growth and their maintenance, including a mimicking of mechanical and biochemical cues, as well as allow for the efficient nutrient transfer, gas exchange, metabolic waste removal, and cell–cell signaling.^113^ Multiple state-of-the-art microfabrication technologies have been used to control the three-dimensional microstructure of SFMA hydrogels, which allows further control over cellular interactions and cell behavior. In this section, we present the most prominent selection of micro- and nanoengineered SFMA hydrogels produced by a variety of additive manufacturing techniques, as summarized in Figure 6. While Figure 6A−F depicts the methods we will discuss, Figure 6G shows the length-scales of structures achieved by these methods.

### 3.1 Micromolded SFMA Hydrogel

Micromolding is one of the most common, simple, and cost-effective methods that can be used for fabricating hydrogel scaffolds with planar and nonplanar surfaces. Using this method, structural attributes such as size, thickness, and pattern can be determined by creating molds.^5^ Molds may be made of different materials, such as metal or plastic, which are commonly referred to as polytetrafluoroethylene (PTFE) or polydimethylsiloxane (PDMS).^114^ PDMS is also one of the most commonly used materials for fabricating micromold replicas.^115^ PDMS has tunable mechanical strength, a transparent optical appearance, and is largely biocompatible, making it ideal for micromolding.^115^ Furthermore, the surface properties of PDMS molds can be altered in order to alter the fluid wettability of liquids and facilitate the easy removal of hydrogels fabricated from the mold.^116^ Furthermore, photo-cross-linking combined with micromolding can be used to form patterned hydrogels from prepolymer solutions, as previously explained.^5^ As described in Barroso’s study, molded hydrogel was fabricated by pouring 200 μL of SFMA hydrogel into a cylindrical mold containing a diameter of 12 mm. Afterward, they were illuminated with UV-A at a light intensity of 3 mW/cm^2^ for 5 min in order to fully cure to retain shape fidelity.^30^

### 3.2 Photopatterned and Shape-Defined SFMA Hydrogels

Since SFMA is a photo-cross-linkable hydrogel, the idea of using a photopatterning technique for the preparation of a hydrogel with a particular topography or for building 3D structures with SFMA-derived hydrogels is appealing. In order to achieve this photopatterning, photomasks can be used, in which the light is irradiated only through the transmittance regions of the photomask and causes a chemical cross-linking to result in spatially confined places resulting in patterned structures. Youn et al. have prepared the micropatterned SFMA/Eumelanin composite films for bioelectronic applications through photopatterning.^36−38^ Furthermore, the lithography technique is one of the advanced methods of fabricating micro- or nano- structures at extremely small scales that allows the creation of precise and complex two-dimensional (2D) or three-dimensional (3D) shapes.^117^ Pal et al. have developed an SFMA biopolymer which can easily be produced with particles of precise shapes (circles/discs, arrows, squares, triangles, stars) in the range of 5 to 500 μm.^36^ They found that SFMA particles produced this way are biocompatible and degrade within several weeks (in 1 U protease in PBS/mg of protein settings). It is also possible to control the rate of degradation through the adjustment of particle size, thickness, and cross-linking levels of SFMA proteins. Hence, by varying particle thickness, size, polymer types, and cross-linking levels, the potential release of encapsulated drugs in such SFMA particles can be altered,^36^ generating future controlled release materials.

### 3.3 Micropatterned SFMA Hydrogels

One method of micropatterning is to create various surface groove patterns using optically graded glass substrates, followed by the casting of PDMS replica molds. This method, however, has a number of disadvantages, including being hard to scale up and not readily reproducing complex designs due to the need to create transfer molds.^118^ Xu et al. developed flexible, strong, micropatterned and biodegradable 2D SF based sheets for cellular adhesion and proliferation.^118^ It was determined that the prepared films are smooth and have patterns ranging between 10 and 25 μm and a pattern height of 500 nm.^118^ On SFMA patterned film, the human bone marrow–multipotent stromal cells (hBM-MSCs) showed the highest proliferation rate and exhibited clear alignment according to the grid pattern design.^118^ A further finding was that the cells adhered to the grids of the organization, and it was even more interesting to observe that the cells could migrate between grids and self-assemble into sheets.^118^ The films can be formed in a variety of thicknesses, ranging from 10 nm to 10 μm thick, with controllable degradability (at 1U protease in PBS/mg of protein).^118^ The strength of the film was determined to be more than 100 MPa.^118^ It was possible to roll, bend, or change the conformation of the film numerous times without losing any of its chemical or physical properties.^118^ By controlling the surface architecture and topography of the flexible sheets, they were able to control the adhesion and spreading of the cultured hBM-MSC cells, showing the potential of this fabrication method for anisotropic tissue constructs.^118^

### 3.4 Bioprinting of SFMA Hydrogels/Inks

The development of 3D printing technology has captured significant attention in regenerative medicine and clinical applications due to its ability to produce scaffolds recapitulating more precise and heterogeneous shapes.^119^ A crucial aspect of this procedure is the development of biocompatible, biodegradable, and printable bioinks. Protein-based hydrogel materials provide an excellent alternative for providing the ECM needed for the encapsulation of cells and the creation of tissues and, indeed, to be used as bioinks. Using the GMA for modification of a SF hydrogel, Kim et al. have developed SF-GMA, which can be used to develop 3D printable bioinks. A biocompatibility study was conducted using NIH/3T3 cells encapsulated in SFMA at different concentrations of 10, 20, and 30%. 10% GelMA was used as a control to compare the biocompatibility of SFMA. In addition, DLP 3D-printing technology was used by Kim et al. for encapsulating the cells without damaging them due to their fast and precise optical printing speeds. On the other hand, they found the swelling behaviors, compressive stress, compressive strain, and compressive elastic modulus at 50% strain, tensile stress, and elongation at break (%) of hydrogel were tuned, and progressively increased, by changing the concentration from 10 to 30%, as shown in Table 3. Based on their results, 30% SFMA resulted in scaffolds that were most easily printed and with best cell growth interactions.

**Table 3:** Analysis of SFMA-Containing Hydrogels’ Mechanical Properties^a^

SFMA content (wt %)	10	20	30
compressive stress at break (kPa)	122 ± 45	434 ± 128	910 ± 127
compressive strain at break (%)	69.5 ± 0.5	77.7 ± 3.3	80 ± 5.1
compressive elastic modulus at 50% strain (kPa)	17.7 ± 0.3	47.8 ± 4.8	125.8 ± 34
tensile stress at break (kPa)	ND	52 ± 4.3	75 ± 7.5
elongation at break (%)	ND	77.6 ± 3.8	124.2 ± 41
tensile elastic modulus at 50% strain (kPa)	ND	9.7 ± 1.0	14.5 ± 2.9
water content at 5 h from dried SFMA (%)	4580 ± 480	2026 ± 202	1059 ± 121
expansion rate at 5 h (%)	175.2 ± 6.1	150.0 ± 0.9	145.4 ± 2.4

a ND indicates no significant difference. The original manuscript used Sil-MA abbreviation, whereas in this paper, we use coherent SFMA abbreviation instead. Reprinted with permission under a Creative Commons CC-BY 4.0 License from ref (24). Copyright 2018 Springer Nature.

### 3.5 SFMA Hydrogel Fibers and Fabrics

Other techniques to produce complex fibers and scaffolds with highly tunable mechanical properties, combined with an interconnected structure include electrospinning and wet spinning. Since the SFMA stabilization requires a UV illumination system during cross-linking in addition to a fiber spinning system, this poses a technological challenge for the efficient fabrication process. In order to address this issue, the researchers electrospun the SF and then modified their surface by grafting GMA and MA onto the SF. Bae et al. have used the SF solution to electrospin mats that were postfunctionalized using GMA chemistry. Functionalization of the SF with GMA can result in dual cross-linking of the resulting fibrous membrane, including physical cross-linking with ethanol immersion by transformation from a random coil to a β-sheet.^34^ Further, this fiber can be cross-linked chemically using radical polymerization to improve its water resistance and controlled degradability.^34^ The total water solubility of fibers in water after dual cross-linking (physical and chemical cross-linking) was much lower, approximately 2%, than that of fibers with only physical cross-linking, approximately 5–7%.^34^

### 3.6 SFMA Nanoparticles

In recent years, hydrogel nanoparticles (NPs), often referred to as nanogels, have gained attention for their potential application in drug delivery systems. Additionally, hydrogel nanoparticle materials display both the characteristics and features that hydrogels and nanoparticles individually possess. These particles are ideally suited for pharmaceutical applications because of their hydrophilic, flexible, versatile, highly water absorptive, and biocompatibility properties. A variety of methods have been used to prepare NPs with hydrogel consistency. Many studies have been conducted on a wide range of methods for manufacturing SF NPs, including emulsification,^120^ salting out,^121^ supercritical CO_2_,^122^ and nanoprecipitation.^123^ Aside from nanoprecipitation, microfluidic synthesis has also been used to optimize control, reduce waste, and improve quality and consistency. Although the above-mentioned method was used in the preparation of the SF nanoparticles, little research has been conducted on the preparation of SFMA NPs. One method of the preparation of the SFMA NPs is a molecularly imprinted polymer nanoparticle (MIP-NP).^29,124^ The Bossi group developed imprinted SFMA NPs that assessed binding sites for conjugation into human serum albumin (HSA). To achieve this, 3 nmol of HSA was added to 4 mL of SFMA at concentrations of 0.3 and 0.03 w/v in the presence of 0.2% w/v LAP. They evaluated the size and polydispersity (PDI) of those SFMA NPs after cross-linking by dynamic light scattering (DLS). The size and PDI for two different concentrations of 0.03 and 0.3 w/v% of SFMA solution were 52.8 ± 0.1 (PDI 0.13 ± 0.01) and 94.6 ± 1.3 nm (PDI 0.36 ± 0.02), respectively. As well, they determined that the mean molecular mass of these SFMA NPs for 0.03 and 0.3 w/v % was 7 and 21 MDa, respectively. Furthermore, they used microfibers (raw microfibers after degumming) and electrospun nanofibers to test the performance of MIP SFMA NPs in mat form to make biomedical textiles. This was accomplished by degumming raw cocoons, dissolving them in formic acid, and electrospinning them to produce a nonwoven tissue with fiber dimensions of 300 ± 75 nm. They compared these nanofibers with raw microfibers control group. Afterward, the rhodamine-conjugated MIP NPs were coupled to both SF microfibers, raw microfiber, and also to SF electrospun nanofibers by addition of acryloxyethyl thiocarbamoyl rhodamine B at 0.02% w/v in DMSO. It was found that the raw SF microfibers did not exhibit the endogenous fluorescence they had expected. Furthermore, the fluorescence signals produced by SF nanofibers in contracts are characterized by a specific and homogeneous fluorescence, which indicates that the SF nanofibers have been uniformly functionalized with rhodamine-conjugated SFMA MIP NPs. According to cytotoxicity results, both 0.25 and 1.5 mg/mL SFMA MIP NPs showed less than 15% cell death (similar to control), indicating they are nontoxic and largely biocompatible. Bossi et al. were the first to propose a SFMA as macromolecular monomer for the purpose of the preparation of the stable forming of the nanoparticles for immobilization of the MIP-alb (human serum albumin immobilized MIP).

## 4 Applications Explored to Date

SFMA hydrogels have been designed and explored for multiple tissue engineering and biomedical applications. In the following sections, we aim to provide the most prominent current state of the art examples of their use, along with links to their physicochemical and structural properties.

### 4.1 Application in Tissue Engineering and Regenerative Medicine

**Figure 7:**
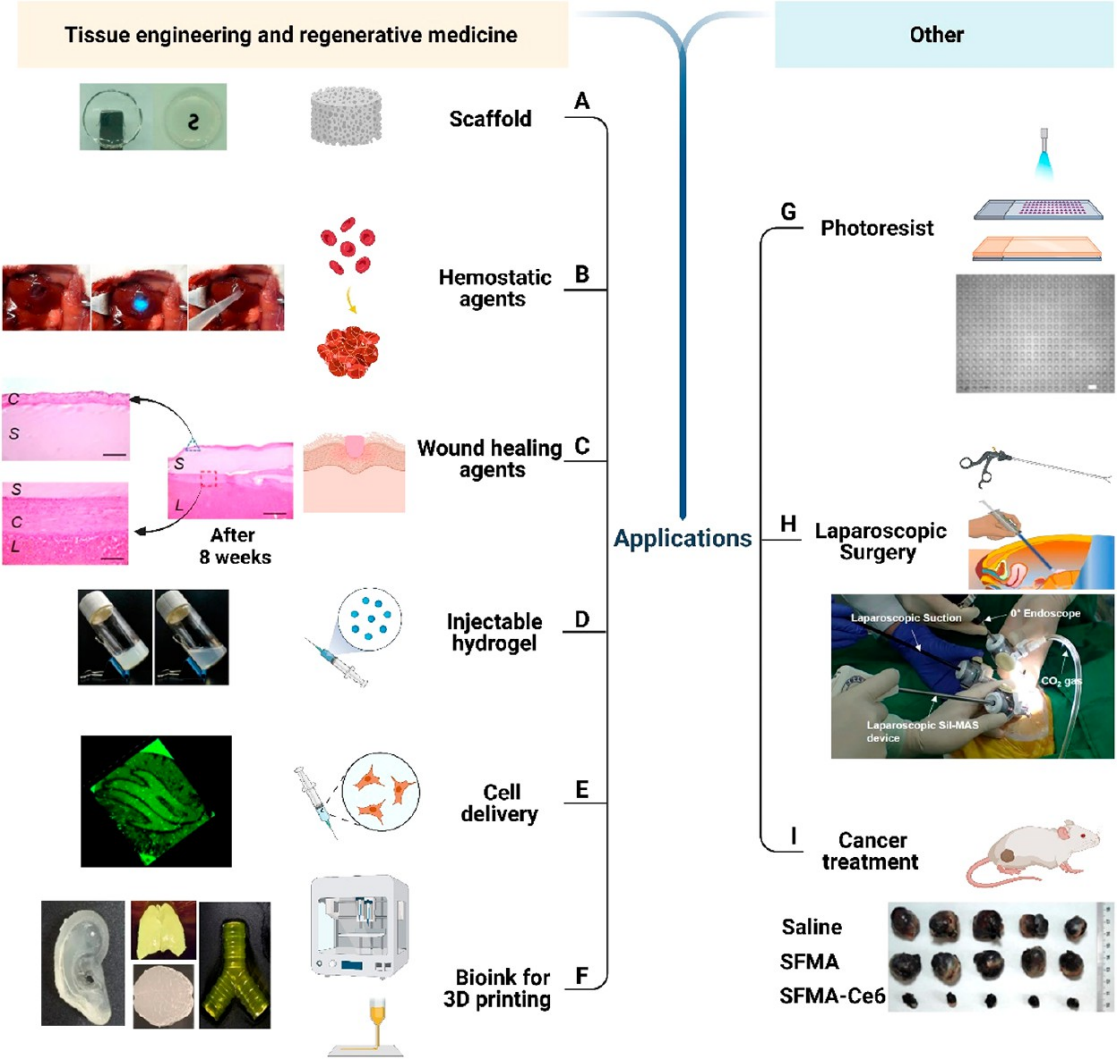
SFMA is designed for use in a variety of biomedical applications, including (A) scaffolds. Reprinted with permission from ref (30). Copyright 2021 American Chemical Society. (B) Hemostatic and (C) wound healing agents. Reprinted with permission under Creative Commons CC-BY 4.0 License from ref (33). Copyright 2020 Springer Nature. (D) Injectable hydrogels. Reprinted with permission from ref (93). Copyright 2021 Wiley-VCH GmbH. (E) Cell delivery and (F) bioink for 3D printing of tissues and organs, including cartilage, lungs, brains, skin, and ears. Reprinted with permission under a Creative Commons CC-BY 4.0 License from ref (24). Copyright 2018 Springer Nature. (G) Photoresists. Reprinted with permission from ref (36). Copyright 2016 Elsevier Ltd. (H) Laparoscopic devices. Reprinted with permission under Creative Commons CC-BY 4.0 License from ref (33). Copyright 2020 Springer Nature. (I) Cancer treatments. Adapted with permission from ref (93). Copyright 2021 Wiley-VCH GmbH. Overall figure was created with Biorender.com, with individual elements used with annotated permissions.

As can be seen in Figure 7 and from previously described sections, SFMA is a versatile biomaterial with a wide range of adaptable physical and chemical properties, which result in compatibility for a wide variety of applications. In the first instance, we will discuss the general cytotoxicity toward the tissue engineering applications by demonstrating types of cells that have been grown and shown to interact with SFMA. As shown in Table 4, the most common types of studied cells include NIH/3T3, L9292, MEFs, human chondrocytes, and A549.

**Table 4:** SF Modified by a Variety of Monomers, Including MA, IEM, GMA, and CA and Their Applications

grafted monomer	degumming approach	polymer named	cells	applications	significant observations	ref
	extraction					
MA	0.02 M Na_2_CO_3_, 30 min	SFMA	3T3 murine fibroblasts	bone tissue engineering	high cell viability (over 95% in 1st and 4th day)	(26)
	9.3 M LiBr				improved mechanical properties (70 kPa for 5.2% SFMA)	
					supporting cell growth	
					tuning the cell behavior with changing hydrogel stiffness and hydrophilicity	
MA	0.02 M Na_2_CO_3_, 60 °C, 30 min	SFMA	B16F10 melanoma cells	antitumor effects	effective photodynamic	(93)
	9.3 M LiBr, 60 °C, 30 min		RAW264.7 cells	skin repair	antibacterial activity	
				hair follicle regeneration		
IEM	0.02 M Na_2_CO_3_, boiling temperature, 30 min	FPP	murine fibroblast (L-929) cells	can be applied in the microelectronics industry and SF-based microdevices	simplicity of the production	(37)
	9.3 M LiBr			functionalization of nanomaterials through biotemplating or prototyping tools	tuning the mechanical strength and degradation	
				biological arrays, tissue engineering, and drug delivery	being biocompatible	
					formation of the complex structure with precise shape and size	
IEM	0.02 M Na_2_CO_3_, 100 °C, 30 min	FPP	female mouse embryonic fibroblasts (MEFs)	can be used for optics, delivery, or bilocally functional agents	monodisperse and precise shapes	(36)
	9.3 M LiBr			controlled release applications	fully biodegradable	
					biocompatible	
					controlling degradation with carrying size, thickness, and degree of cross-linking of SF proteins	
IEM	0.01 M C_18_H_33_NaO_2_	SFMA		various biomedical applications, 3D printing ink, injectable hydrogel, cell culture matrix and surgical glue.	good water solubility at various degrees of methacrylation	(35)
	0.02 + 0.02 M Na_2_CO_3_, 60 min				quick gelation time (<60 s)	
	9.3 M LiBr: 0.6 M NaOH, 80 °C				high elasticity compared to physically cross-linked SF	
GMA	0.05 M Na_2_CO_3_, 100 °C, 30 min	silk-GMA	NIH/3T3 cells	cartilage regeneration	capability to be used as bioink	(73)
	9.3 M LiBr, 60 °C, 1 h		human chondrocyte	3D DLP printing	good mechanical strength	
					good biocompatibility	
GMA	NM	SF-GMA		scaffold and wound dressing agents	dual cross-linking mechanism can be used	(34)
	CaCl_2_/EtOH/H_2_O (1:2:8 M ratio), 70 °C, 1 h				high stability for cell attachment, migration, and proliferation	
GMA	0.05 M Na_2_CO_3_, 100 °C, 30 min	SB	Neuro2a cells	digital light processing (DLP) printable bioink	high mechanical strength (>500 kPa)	(28)
	9.3 M LiBr, 60 °C, 1 h				electroconductivity increased by incorporation of GO in SFMA	
GMA	0.05 M Na_2_CO_3_, 100 °C, 30 min	Sil-MAS	NIH/3T3 cells	*in vitro* and *in vivo* hemostatic and wound healing effects	sealing without any surgical method	(33)
				laparoscopic tool in field of robotic surgery	excellent adhesive properties with wound closure strength more than 25 kPa	
	9.3 M LiBr, 60 °C, 1 h			versatile medical glue for clinical applications	hemostatic effects	
					high biocompatibility	
					adequate degradation time (25.1% *in vitro* degradation in 30 days)	
GMA	0.02 M Na_2_CO_3_, 100 °C, 1 h	Sil-MA	primary meniscus cell (pMCs)	an attractive alternative to producing fibrocartilaginous tissues.	excellent structural integrity and biomechanical performance	(27)
	9.3 M LiBr, 70 °C, 1 h				integrity, with no dimensional changes to fibrocartilaginous tissue	
GMA	0.1 M Na_2_CO_3_, 100 °C, 30 min	SF-*g*-GMA		meniscus tissue engineering	effect of the CaCl_2_ concentrations and ratio of the SF to the CaCl_2_ on SF solubility	(125)
					effect of the dialysis time on efficiency of the salt removing	
	3.6, 4.5, and 5.4 M CaCl_2_, 70 °C, 6 h				effect of the SF to GMA molar ratio on grafting process and amounts	
GMA	0.05 M Na_2_CO_3_ 100 °C, 1 h	silk-GMA	NIH/3T3 cells mouse embryonic fibroblast cell line	hydrogel with potential to be applied in clinical transplantation for tissue engineering and biomedical applications	effect of dialysis period on the β-sheet contents of SF and cell proliferation and viability rate (live–dead assays showed 66% and 97% cell viability for hydrogels with 0 and 7 days of dialysis)	(60)
	9.3 M LiBr, 60 °C, 1 h					
GMA	0.02 M Na_2_CO_3_, 100 °C 30 min	silkMA	human dermal fibroblasts (HDFs)	tissue engineering applications	the effect of the pH on the rheology of the hydrogel, mechanical strength, swelling behavior, cell proliferation and attachments; the SFMA with pH 8 shows more than twice hydrogel expansion (34.4%) and swelling ratio (86%), compared with SFMA with pH 5	(30)
	9.3 M LiBr, 60 °C, 1 h				the compressive modulus of SFMA at pH 5 is twice as high (40 kPa) as SFMA at pH 7	
					cell viability of more than 90%	
GMA	1st bath: 0.01 M Na_2_CO_3_ 100 °C, 1 h	SF-MA	NIH 3T3	can be used as biomaterials with extra functionalities	nontoxic	(29)
	2nd bath: 0.001 M Na_2_CO_3_, 100 °C, 1 h				biocompatible (more than 80% cell viability)	
	9.3 M LiBr, 4 h, 65 °C					
GMA	0.02 M Na_2_CO_3_, 100 °C, 1 h	Sil-MA	NIH 3T3	Ink to build complex organ structures, including the heart, vessel, brain, trachea and ear	tuning the mechanical strength with varying the SFMA content (compressive stress at break of around 910 kPa for 30% SFMA)	(24)
			chondrocytes were isolated from human septal cartilage		outstanding mechanical and rheological properties	
	9.3 M LiBr, 60 °C, 1 h				excellent structural ability	
					reliable biocompatibility	
CA	0.03 M C_18_H_33_NaO_2_	SF-NB	mouse embryonic fibroblasts (NIH/3T3)	three-dimensional cell culture requiring temporal control of hydrogel stiffness	controllable cross-linking based on two mechanisms including thiol–ene and photoclick reaction.	(39)
	0.04 + 0.02 M Na_2_CO_3_ 60 min			cell encapsulation	elastic moduli of 1.5–3 kPa depend on the SFNB contents (0–4 wt %)	
	9.3 M LiBr 60 °C, 1 h		adenocarcinoma human alveolar basal epithelial cells (A549)	tumor development and tissue fibrosis model		
CA	0.05 M Na_2_CO_3_, 100 °C, 40 min	RFS-NB	mouse fibroblast (L929) cells	3D printed scaffolds for tissue engineering	good biocompatibility (>95%)	(40)
	9.3 M LiBr, 40 °C, 40 min				mechanical strength depends on the UV exposure time (by increasing the UV exposure time from 1 to 10, the ultimate storage modulus increases from 51 to 1700 Pa)	

Recent reports have demonstrated that SFMA hydrogels can be effectively used as an active matrix material in both 2D^30^ and 3D^24^ cell culture experiments. To form cell-laden 3D hydrogels, it is possible to suspend cells in SFMA prepolymer solutions and cross-link them upon exposure to UV light. According to Kim et al., NIH/3T3 cell viability and proliferation in SFMA hydrogels changed as a function of the UV cross-linking time. The samples were exposed to UV for 10, 20, and 30 s, and it was found that the samples with longer irradiation times (20 and 30 s) demonstrated a higher cell proliferation ratio than those with lower levels of irradiation or pristine SF.^33^

Barroso et al. evaluated the cell attachment and proliferation with direct seeding of human dermal fibroblasts (HDF) cells on different types of SFMA (prepared at three different pH values of 5, 7, and 8) and compared it to the GelMA hydrogel, which due to its extensive studies and use in cell culture applications constitutes a benchmark. Based on these results, it appears that SFMA hydrogels are capable of supporting HDF cell attachment and proliferation; however, the cells deposited in SFMA hydrogels showed relatively little spreading compared to the GelMA hydrogels. These reasons may be attributed to the lack of adhesive amino acid sequences (e.g., RGD) in the SF composition. Although, the results show that after the cells had been grown for 7 days, they exhibited typical fibroblastic morphology and reached confluence, similarly to the results observed on GelMA hydrogels.^30^

As previously mentioned, an elastic hybrid construct for advanced fibrocartilaginous tissue regeneration using GG/FB composite bioinks in conjunction with SFMA bioink hydrogels was developed by Costa et al.^27^ This formulation of SFMA 16% (w/v) contained gelatin and glycerol with concentrations of 4.5% (w/v) and 8 (w/v) %, respectively. A GG/FB bioink contains GG with a fixed concentration of 12 mg/mL and FB with concentrations ranging from 1, 2, 3, 4, 5, and 6 mg/mL. Compared to other concentrations, the GG/FB4 was considered the best GG/FB bioink *in vitro* and *in vivo*. In this study, a 3D integrated tissue-organ printing system was used for the 3D printing of the constructs. Furthermore, the bioinks GG/FB and SFMA were printed under two different conditions. GG/FB bioinks were printed using a nozzle with a diameter of 240 μm, at velocity of 250 mm/min, and air pressures between 45 and 65 kPa. Following 3D printing, the constructs were cross-linked with thrombin solution (20 U/mL) for 30 min. In contrast, SFMA bioinks were printed at 250 mm/min, with 300 μm diameter nozzle, and with 450 to 550 kPa air pressure.^27^ Their results showed that the SFMA bioinks offered biomechanical properties and structural integrity, while the GG/FB bioink offers a microenvironment that ensures the maintenance of cell viability and proliferation.^27^

Using a standard digital lighting processing (DLP) printer, Hong et al. encapsulated and printed the NIH/3T3 mouse fibroblast cells and human chondrocytes in SFMA bioink hydrogel for cartilage tissue engineering. As part of their study, they evaluated the chondrogenesis of human chondrocyte-laden SFMA *in vitro* and then applied it *in vivo*. The authors ensured even cell distribution in printed SFMA hydrogels due to the rapid printing speed and photopolymerization in DLP method utilized here. The contents of glycosaminoglycan (GAG) in human chondrocytes-laden SFMA were evaluated after cultivation for 1, 2, and 4 weeks to determine whether *in vitro* chondrogenic regeneration had occurred. While the amount of GAG did not change significantly over the first week, after 4 weeks, it had remarkably increased, about three times the amount in the first week. They also examined the expression of cartilage specific genes such as collagen type II, collagen X, SOX-9, and aggrecan in cultured 3D chondrocyte cells laden-SFMA using reverse transcription-polymerase chain reaction (RT-PCR) and gel electrophoresis. During the first and second week of culture, the collagen type II, collagen X, SOX-9, and aggrecan genes were not expressed. After 4 weeks of cultivation, these genes were expressed, indicating SFMA bioink containing chondrocyte cells could lead to generation of functional cartilage with SFMA.^73^

### 4.2 Other Therapeutic Applications

*In situ* formed hydrogel offers inherent advantages for a wide range of biomedical applications such as oncology, drug delivery, and tissue reconstruction. The input factors of local microenvironments (temperature, pH, enzymes, etc.) or external factors (light) enable injectable precursor solutions to directly fill irregular defects. The studies confirmed that the SF-based hydrogel had been found to be biocompatible in biomedical applications without causing any long-term inflammatory reactions.^14^ Additionally, the research done previously indicates that SF offers hemostasis, which is very beneficial to wound healing progress.^126,33^ In addition, it promotes the recruitment of various types of cells, such as neutrophils, macrophages, endothelial cells, fibroblasts, and keratinocytes, and facilitates the proliferation of skin fibroblasts.^93^

As previously mentioned, Tang et al. have demonstrated the potential of using hydrogels derived from SFMA and chlorine e6^93^ to enhance wound healing and cancer treatment. Following previously described successful *in vitro* study (see section 2.3), the authors have further evaluated the efficacy of SFMA-Ce6 hydrogel *in vivo*. This was accomplished by creating 5 mm wounds at tumor sites on BALB/c nude mice bearing B16F10 tumors. The authors assessed the therapeutic effects of *in vivo* photodynamic therapy by covering tissues with saline (control), SFMA (SFMA-20), or SFMA-Ce6 hydrogel and exposing them to NIR (660 nm, 20 mW cm^–2^) light for 10 min. After photodynamic therapy, tumor volumes in the SFMA-Ce6 group were significantly lower than those in the saline or SFMA groups, and wound closure was better in SFMA-Ce6 mice than in the other groups. It can be concluded that this silk-based hydrogel system, as a wound dressing that is multifunctional, paves the way for new techniques of tumor therapy and skin regeneration for humans.^93^

**Figure 8:**
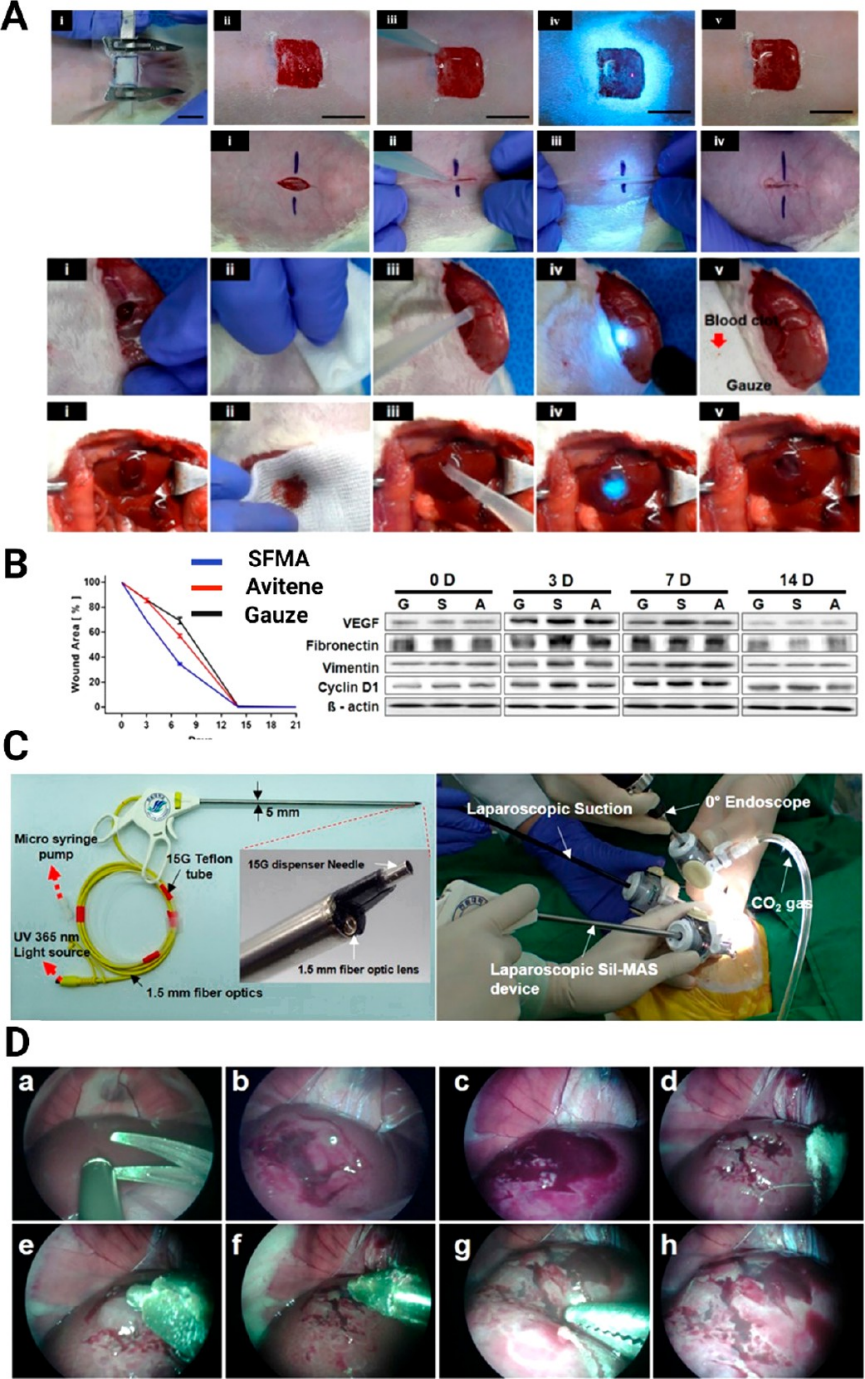
Illustration of (A) the hemostasis measurement on a skin defect in a rat (1 cm (w) × 1 cm (d) × 0.3 cm (h)), wound closure test with skin incisions, 1.5 cm length, *in vivo* vascular closure test, *in vivo* rat liver parenchymal injury model, using SFMA. (B) The wound area after dressing treatment with SFMA, along with a Western blot analysis of VEGF, fibronectin, vimentin, cyclin D1, and β-actin shows the strongest expression at day 3, and (C) laparoscopic surgical procedure images of a liver laceration model with a homemade SFMA device and a three-pot laparoscopic field. (D) (a–c) Create deep and superficial liver lacerations with endoscopic scissors and suction, (d) apply gauze, (e) administer 1 mL SFMA by using a homemade laparoscopic SFMA device, (f) expose to UV light (20 s) via fiber optic lens, (g) monitor for blood leakage or rebleeding with endoscopic forceps, and (h) ensure the SFMA adheres to the liver laceration. The original manuscript used Sil-MA abbreviation, whereas in this paper, we use coherent SFMA abbreviation instead. Reprinted with permission under a Creative Commons CC-BY 4.0 License from ref (24). Copyright 2018 Springer Nature.

Surgical sealants are designed to prevent gas leakage or nonclotting fluids from the injured tissues and blood from the vascular supply after surgery or injury.^127^ A surgical sealant has also been made with SFMA hydrogels as another exemplar application of this material. Kim et al. propose that SFMA could be a suitable photocuring medical glue used to improve laparoscopic surgery.^33^ As part of the preparation of the SFMA, GMA was used to functionalize SF. A 25% (w/v) concentration of SFMA hydrogels was prepared and tested for their properties, including compression strength, tensile strength, rheological properties and adhesive properties. Kim et al. compared SFMA 25% to the commercial liquid bandage and active sealant Medifoam (MLB, Mundipharma). It was observed that the shear strain and shear stress at the break of SFMA were higher than those of MLB. Further, the adhesive strength of SFMA was strong enough to hold and lift a wrench (0.9 kg). Furthermore, to investigate the wound closure properties of the SFMA, the researchers made skin incisions 1.5 cm long on the backs of the rats. After applying 200 μL of SFMA onto the wound, they exposed it to UV light for 10 s in order to cross-link it, as shown in Figure 8A. In comparison to gauze, SFMA, and Avitene (commercial artificial dermis Avitene), the SFMA treatment showed a lower wound area and a better wound closure (Figure 8B). Moreover, they also tested SFMA sealant properties on an animal model of femoral artery hemorrhage. This experiment involved the creation of an incision in the femoral vein and artery using a sterilized scalpel. The injured area was gently covered with gauze for 3 s following the initial 5 s of bleeding. Subsequently, 50 μL of SFMA were applied to the area and exposed to UV light for 20 s, Figure 8B. Additionally, they examined the biocompatibility and degradation of the SFMA *in vitro* via the CCK-8 assay and *in vivo* using subcutaneous hydrogel implants.^33^ They also exposed the SFMA to UV radiation for 10, 20, and 30 s, and then compared it with SF (pristine). According to the CCK-8 results, there was no significant difference in cell viability between SF and SFMA with different exposure times on day 0. It should be noted that after 3 days of cultivation, the sealant that was exposed for longer times, 20 and 30 s, had a greater level of viability than the sealant which was exposed for shorter times (10 s). In the long term, SFMA increased the number of vascular structures and collagenous tissue while decreasing the number of infiltrating macrophages, reflecting its biocompatibility. Based on the *in vivo*, SFMA has proven to be effective at providing secure and long-lasting sealing due to its excellent adhesive properties, hemostatic properties, and wound healing properties.^126,33^ The SFMA also demonstrated good biocompatibility and long-term degradation, so it was not necessary to retreat or remove it from the injury site.^33^

Based on the results of Kim et al., it appears that SFMA can be used as photocuring medical glue during laparoscopic surgery. They developed an endoscopic device composed of SFMA and a light source, which is used for laparoscopic surgery (Figure 8C). As shown in Figure 8D, the laparoscopic surgical device was used to administer the SFMA, and the UV treatment led to successful gelation, adhesion, and hemocoagulation of the liver tissue.^33^ Hence, SFMA is a viable option to enhance the efficiency of laparoscopic procedures, proving itself as potential translational surgical glue.

## 5 Conclusions and Future Perspectives

SF exhibits a number of characteristics that distinguish it from other materials, including its biocompatibility, biodegradability, nontoxic nature, mechanical strength, and ease of acquisition.^128^ Despite the fact that SF is soft in nature, its potential applications are limited by this characteristic.^128^ By introducing functional groups such as methacryloyl and norbornene, the cross-linkable sites on the SF are created. Here we discussed several approaches to achieving these in SF, including influence of its original sources and roadmap from degumming and extraction, to final modification with methacrylate and norbornene. Moreover, we described different approaches to methacrylating silk fibroin using different methacrylation reagents, including methacrylic anhydride (MA), 2-isocyanatoethyl methacrylate (IEM), or glycidyl methacrylate (GMA) and norbornene groups. We highlight that SFMA and SFNB hydrogel storage moduli may differ depending on the SF source, the degree of modification with functional photo-cross-linked group, and the initiator concentration, as well as the UV intensity and other environmental factors including pH or type of used solvent.

SFMA’s and SFNB’s photo-cross-linkable biomaterials have been additively manufactured using microfabrication methods, including photopatterning,^36^ micromolding,^30^ and DLP bioprinting,^27,28,24^ which can also be employed for encapsulating cells *in situ* in hydrogel with a defined 3D structure and topology. Although some fabrication techniques have been used, we highlight that to our knowledge the extrusion-based printing of SFMA-based materials has not been efficiently achieved or reported.

Finally, we summarized the SFMA and SFNB-based bioinks and their composites in terms of their potential for the use in regenerative medicine and tissue engineering. We anticipate that new microfabrication techniques such as microfluidics and wet-spinning will enable SFMA-based materials to be used in a wider range of applications, e.g. in form of the microcapsules and microfibers, in the next few years. Nevertheless, there are still a number of improvements that must be made in the formulations and characterization of inks in order to make *in situ* 3D printing a reliable process.^43^ It is crucial to standardize SF extraction protocols, examine exact *in vivo* degradation mechanisms, and set sterilization protocols compatible with clinical conditions.^43^ SF sequences can be easily chemically modified, which may play a crucial role in possibilities of using them as controlled materials or for the binding of specific biomolecules to them. It is important to note, however, that the extracted SF solution is stable only at a specific pH and temperature ranges.^129^ Consequently, it is important to take this point into consideration when binding growth factors or enzymes to SF-based biomaterials, or when using these *in vivo*.

Given so far the established properties of SFMA, it certainly carries the potential to be used as a cargo vehicle for the delivery of bioactive agents, such as anticancer drugs, anti-inflammatory drugs, or growth factors, and numerous chemical groups on it will enable an environment of controlling the release properties by tuning of molecular interactions, as is the case for other peptides or protein-based materials.^130^ Moreover, this photo-cross-linkable SF-based hydrogel has the potential to be developed into a functional biomaterial that is capable of sealing (and even healing) surgical injuries, which could lead to the development of biomaterials that in future release oxygen in the wound during healing process. Thus, various formulations of SF-based bioinks need to be evaluated, characterized, and standardized in order to prevent all potential *in vivo* cytotoxic effects of the photoinitiators. Further, a number of improvements must still be made before *in situ* systems can be developed to address tissues mechanical, cellular, vascular, and innervation requirements.
